# Nesting niche partitioning between two sympatrically breeding *Chlidonias* Tern species revealed by remote sensing

**DOI:** 10.1038/s41598-025-06205-4

**Published:** 2025-07-01

**Authors:** Karolina Cieślińska, Brygida Manikowska-Ślepowrońska, Krzysztof Ślepowroński, Dariusz Jakubas

**Affiliations:** Department of Vertebrate Ecology and Zoology, Faculty of Biology, University of Gdańsk, Gdańsk, Poland

**Keywords:** Colonial nesting, NDWI, NDVI, Sympatric nesting, UAV, Wetlands, Zoology, Ecology, Conservation biology, Wetlands ecology

## Abstract

Sympatrically breeding avian species may have similar environmental requirements for nesting sites, resulting in interspecific competition. It may be reduced by partitioning resources in space or/and time, allowing relatively stable coexistence in the shared habitat. Here, we investigated nesting niches of sympatrically breeding, Black Terns (BT, *Chlidonias niger*) and Whiskered Terns (WT, *Chlidonias hybrida*) in Druzno Lake (Poland) in 2024. We compared nesting site proximity characteristics between both studied species at two different spatial scales using remotely sensed from drone surveys: (contribution of water and vegetation) and indices derived from satellite imagery: Normalized Difference Vegetation Index (NDVI) as a proxy for vegetation density, and Normalized Difference Water Index (NDWI) as a proxy for open water contribution. Both species partitioned location (they bred in separate locations) and resources within common breeding ground. BT’s nests were situated significantly closer to the lake shore (mean ± SD 102.25 ± 62.47 m) and further away from each other (mean ± SD 22.22 ± 14.81 m) compared to WTs (mean ± SD 257.24 ± 109.64 m & 11.24 ± 10.67 m, respectively). BTs, unlike WTs prefer to nest in areas with higher open water contribution and lower vegetation density. The nesting niches of BT were wider than those of WT, suggesting that BT use more diversified habitats for breeding.

**Supplementary Information:**

The online version contains supplementary material available at 10.1038/s41598-025-06205-4.

The competitive exclusion principle states that coexistence of ideal competitors is not possible^1^. In natural ecosystems, species with similar environmental requirements, i.e., strongly overlapping ecological niches compete over habitat resources^1,2^. Intensive interspecific competition may lead to eviction of one species from the habitat or replacing original native species from local site by dominant, stronger competitor with a greater adaptative plasticity^1,3^. Especially species with a wider niche (generalist), can replace the one with a narrower niche (specialist) in local habitats^4^. Generalists are characterized by higher adaptive plasticity, which often allows expansion of their range due to constant developing novel associations within habitat, while specialists strictly depend on restricted resources^5^.

One strategy which reduces intensity of this interspecific competition is niche partitioning, i.e., a differentiation of used habitat resources between competitor species^1–3^. Growing differences between competitors result in reduction of the degree of niche overlap. Partitioning can occur at different levels. First, resource partitioning is displayed by exploiting different environmental resources (no overlap between the two sides of the conflict) (e.g.^6,7^), or overlap exist only for preferred resources and not for the secondary ones (e.g.^7,8^). Spatial partitioning expressed by occupying different parts of the same habitat, temporal – by being active on different part of season or day^9^, or spatio-temporal – by using the same resources at different location and time^10–13^. Therefore, partitioning in multiple dimensions may maintain the mutual coexistence of similar species in common ecosystem^13^. Niche partitioning and overlap is most often studied in context of foraging^2^ but can also occur in context of nesting habitat, which can be defined as sum of different requirements and parameters of habitat/environment in the proximity to nesting sites (e.g^14^). Strong niche overlap is expected in sympatrically breeding species of the same genus (e.g^15^) due to high similarities of ecological needs.

In this study we investigated nesting niches width in the two species of marsh terns—Black Tern (*Chlidonias niger*; hereafter BT) and Whiskered Tern (*Chlidonias hybrida*; hereafter WT) breeding sympatrically in a hypertrophic lake Druzno in N Poland. Both species are similar in size^16^, breed in similar habitats, i.e., in shallow inland freshwater bodies with floating vegetation (nympheids), have similar diet (insects, small fish, and amphibians)^17,18^, and can form uni-species and/or mixed colonies^19–21^. This study area is a good site to investigate niche partitioning between the studied species for many reasons. Firstly, WTs started to breed there only in the middle of 1990th^22^, as they colonized Eastern and Central Europe during the mid-20th century probably after breeding habitat destruction and collapse of large colonies in Central Asia (between the Volga River, the Ural and the Aral Sea)^19^. BTs, on the other hand, have been nesting in this location since the second half of the XIX century^23^. Secondly, unpublished data from the 2000s revealed common breeding of both species in the study area in mixed colonies^20^. Finally, since the beginning of the XXI century, decline in population of BT with simultaneous expansion of WT has been observed not only at the study site^22^, but also at continental scale^18,19,24–26^. The reasoning behind those differences remains unclear but it is thought to be related with conditions on the breeding grounds leading to different breeding success (e.g.^27,28^), e.g., due to absence of optimal nesting medium^27^, human disturbances in proximity to tern’s colonies^29^, or deterioration of prey item availability/quality for offspring^17^. It cannot be excluded that in some areas, WT, while expanding its range, is competitively superior and may locally displace native BT.

To describe nesting niches and colony parameters of two marsh tern species nesting sympatrically we considered the following variables: (1) spatial including location of the nests in the studied area, and (2) resource/habitat including properties of nests and their surroundings at different scales to nest – close proximity (2-m), i.e., contribution (proportion) of water and vegetation (e.g.^30,31^) and further proximity (7-m) ) a proxy of vegetation density i.e. Normalized Difference Vegetation Index (NDVI)^32^ and open water contribution, i.e. Normalized Difference Water Index (NDWI)^33^, and also distance from nests to lake shore, the neighbouring nests, and fleet active waterway. To investigate nesting site selectivity of studied tern species, we compared values of NDVI and NDWI and measured distances between nesting sites and random points selected from the area of the Druzno Lake (potential breeding area).

We formulated some expectations regarding the studied species:

1) given that both study species share several traits, e.g., common environmental requirements like similar diet or nesting habitat (e.g.^17–20^), and have similar breeding biology (e.g.^18^), and that not all resources are limited in rich habitats (here hypertrophic lake with lush vegetation), we expect that the nesting niches of both investigated tern species overlap at least partially and that at least some individuals of both species nest in common colony (what have been documented in some previous studies, e.g.^19–21^).

2) given that colonies develop and change with progress of the breeding season, we expect decrease in distances between nests and shoreline with progress of the breeding period due to expansion of colony area, and nesting niche expansion with advancement of breeding season because of forming new nesting sites in wider selection of habitats, also including suboptimal locations. Such areas may include use of the peripheral zone of the colony, locations further from the centre and near to open water zones. The use of a wider range of nesting habitats in the late breeding season is a result of the late arrival of birds on nesting sites and secondary broods.

3) given reported interspecies differences in nesting habitat (WTs can nest at open water surface or on floating leaves, unlike the other *Chlidonias* species, which need a foundation for their nests^19^), we expect at least partial nesting habitat partitioning between the studied species.

4) given that both studied species exhibit some habitat preferences with BTs preferring open water areas close to the breeding grounds (e.g.^34^), while WTs preferring areas with floating vegetation that supports their nest platforms (e.g.^35^), we expect that the studied species exploit at least partially different types of habitats (differing in e.g., primary productivity, vegetation/water area contribution) when breeding sympatrically.

5) given a history of both terns in the study area (longer breeding of BTs and recent expansion of WTs, we expect higher adaptiveness plasticity and wider generalist niche of WT compared to narrower niche of more specialized BT. Considering the fact that WT is expanding its range, we expect its larger breeding habitat plasticity^19^ compared to BT breeding in the study area since the XIX century^22^.

## Methods

### Study area

**Fig. 1:**
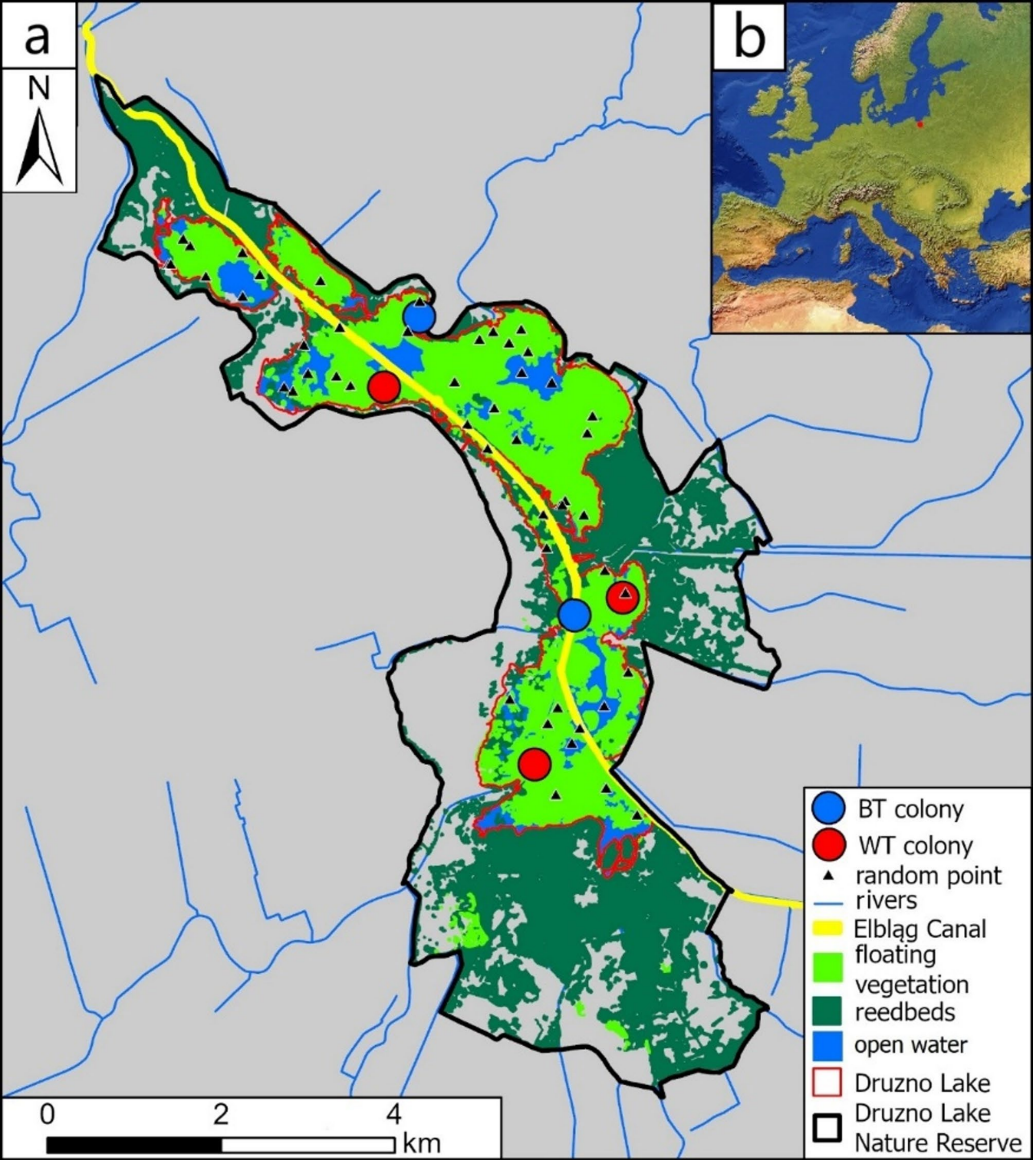
(**a**) Druzno Lake Nature Reserve area (black outline) with highlighted Druzno Lake (red outline), BT and WT colonies (blue and red points, respectively), randomly sampled points (black triangles), floating vegetation, open water areas, reedbeds, and Elbląg Canal (waterway) and (**b**) location of the study area in Europe [inset map]. Base map source: polygons depicting area of Nature Reserve, its vegetation and the Druzno Lake border were prepared by authors, based on ortophoto map product from geoportal.pl. Inset map source: Natural Earth. Free vector and raster map data (naturalearthdata.com, accessed on 02 January 2024). Maps were produced in ArcGIS Pro ver. 3.2.2 (Esri 2024).

This study was conducted in the Druzno Lake Nature Reserve, the area of which is designated as a RAMSAR and a Natura 2000 site. The Druzno Lake is a shallow (mean depth is 1.2 m) and hypertrophic body of water^36,37^ in Northern Poland (54.09°N, 19.47°E), located in alluvial plain of the Vistula River (Fig. 1). The area of the lake is highly variable due to changes in water levels, covering from 1,300 to 2,900 ha^22^. Water level of the lake fluctuates considerably (water level changes up to 1.5 m), e.g., due to local heavy rainfall and water levels in the Vistula Lagoon directly connected to the Druzno Lake^22,36^. The highest water level during breeding season is present usually in July, while it is the lowest in May/June^36^. Water level fluctuations may pose a treat for birds nesting in lake vegetation, including rushes, reedbeds, and nympheids^38^. The lake surface is covered mostly by nympheids, including White water-lily (*Nymphaea alba*), Yellow water-lily (*Nuphar lutea*), and Fringed water-lily (*Nymphoides peltata*)^39^. Emerged parts of vegetation serve as a nesting site for many bird species, including two species of tern genus *Chlidonias*, i.e., BT and WT^22^.

Breeding BTs has been recorded in the Druzno Lake since the second half of the XIX century^23^. Throughout the XX century the local population has underwent great fluctuations with estimated maximally at ~ 300 pairs breeding in several colonies in 1979 to ~ 15 pairs a decade later^22^. WT first appeared in this location in 1984, and the first breeding attempt of this species was recorded in 1996^22^. After its first records, the species underwent great expansion and growth of the breeding population^22,26^. Since the beginning of the XXI century, BT population started to gradually decline with simultaneous growth of WT population. Breeding BT is considered native to Europe, while WT was originally breeding in the southeast part of Europe and central Asia, but due to loss of breeding habitats, the species range shifted towards west^19^. Decrease of BTs with simultaneous expansion of WTs^18^ has been observed not only locally but also on a national^19,25^ and worldwide scale^19,24,26^.

### Fieldwork

We performed this study in the breeding season 2024. To find active colonies, we surveyed the whole area of the Druzno Lake (Fig. 1) by boat, starting from sites where colonies have been noted before. After finding a colony, we approached it and maintained 20–100 m distance to it to lower stress and disturbance to the birds breeding in the colonies and to avoid presence of boat within aerial surveyed area which could lead to later analysis biases. In total, we conducted six controls from the mid-May until the end of July 2024 (18 May, 4 and 26 June, 8, 19, and 29 July). During the 1st visit we observed one pair of BT but we did not find any signs of formed colonies. During the 2nd visit, we observed colonies of both studied tern species (two for each species). After the 2nd field visit, we observed only WT colonies. Colonies of BT were flooded due to hight water level (authors’ observations), and only single nests were found scattered outside of colony range that we did not consider in analyses. Thus, data collected during visits from the 3rd to the 5th included only WT colonies. Consequently, we estimated temporal changes (between the 2nd and the 5th visits) in width of nesting niches and colony parameters solely for WT.

### Aerial surveys and drone imagery preparation

**Fig. 2:**
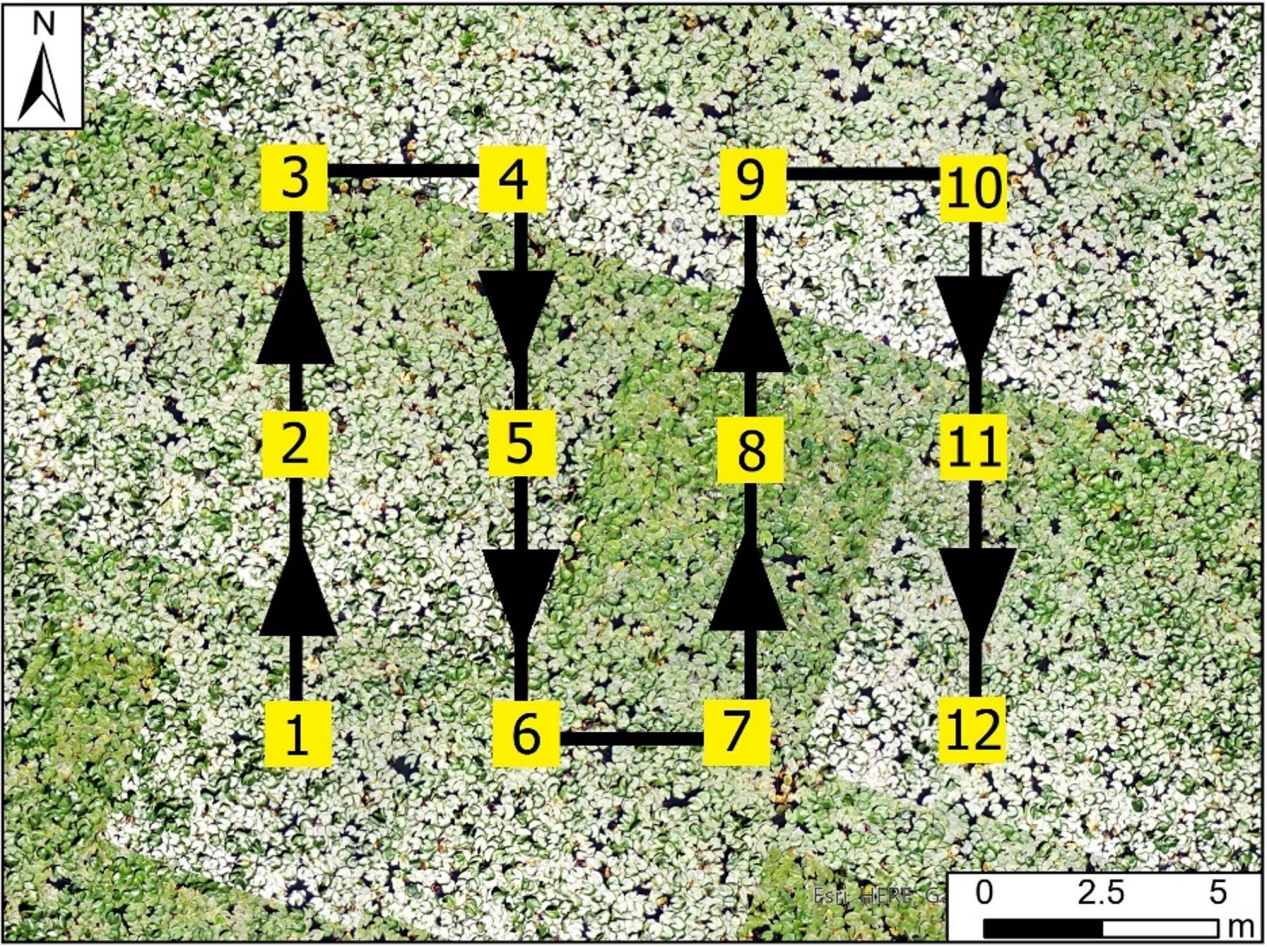
Graphical representation of an example of used ‘lawnmower’ flight pattern. Black line exhibits flight route of UAV, black arrows show direction of flight (drone movement), and numbers in yellow squares indicate following drone positions where photos were taken. Distance between following camera positions is equal to ca. 5 m. Background photo source: photo mosaic from drone. Map was produced in ArcGIS Pro ver. 3.2.2 (Esri 2024).

We performed aerial surveys in all detected colonies using an unmanned aerial vehicle (UAV) DJI Mavic 2 Pro (DJI, China) equipped with a 20 Mpix camera [generic sensor type; L1D-20c model; (FOV: ca. 77°35 mm; Format Equivalent: 28 mm; Aperture: f/2.8-f/11; Shooting Range: 1 m to ∞; Electronic Shutter Speed: 8 –1/8000s; ISO Range: 100–3200) Hasselblad; Sweden] (hereafter a drone). Taken images were saved in both JPG and DNG formats. During flight the drone followed a ‘lawnmover’ (boustrophedonic) motion pattern^40,41^ over the area of interest maintaining the same altitude: 10 m above ground level (see example route in Fig. 2). During each mission, the drone took images (standard RGB multiband products) with gimbal position set to -90° (nadir position) with a 5-meter spatial interval. This way images overlapped with each other at front and sides (ca. 60%< ), which allowed us to successfully obtain orthophoto mosaic product in ArcGIS Pro software ver. 3.2.2 (Esri 2024). We started taking images before entering areas with nests to maximize the effectiveness of nest captures located at the boundary within the generated orthophoto mosaic. In the case of scattered (e.g. present on both sides of Elbląg Canal) and larger colonies (> 20 nests), we performed up to three separate drone flights. Birds quickly adapted (usually < 5 min of the first flight) to presence of the drone performing a regular pattern, but we still conducted drone flights as short as possible to minimize any disturbances at the colony (min-max; mean ± SD: 8–33; 19 ± 7 min). The chosen altitude allowed us to obtain clear images of the colonies and details of their surroundings were distinguishable. Aerial images were later processed in Ortho Mapping workplace in ArcGIS Pro software ver. 3.2.2 (Esri 2024). To avoid misidentifications of nesting sites with uniquely distributed complexes of vegetation, in analyses we excluded cases of incomplete nests with no signs of breeding attempt. For analysis we included active nesting sites [cases of finished or nearly finished nest constructions especially those with visible nesting basins / flattened nest cups visible on vegetation during incubation, cases of nests with visible eggs, chicks, incubating adults, and abandoned nests with visible signs of nesting success (e.g. nearby surroundings intensively covered in guano)].

Drone surveys revealed that BT built small nests on mud mounds, muddy and grassy moveable islands, often with Water soldiers (*Stratiotes aloides*) and dead vegetation (nests built on a ready foundation). Leaves of nympheids were present around the nests but did not constitute a part of the nest foundation. WT on the other hand, built nests on platforms created from fresh vegetation, mostly nympheids. Those platforms always were created on water-lily beds, mostly made from big leaves of White water-lily, but also Yellow water-lily. Both nympheid species were present in areas of two tern species colonies, additionally the Fringed water-lily was present close to some WT colonies and was not observed near colonies of the second species.

We adjusted every cluster of taken pictures, belonging to a single colony by block adjustment tool (we applied the following settings: perform complete camera calibration – refinement of: the focal length of the camera lens; principal point of the autocollimation; radial distortion coefficients (K1, K2, K3); and tangential distortion coefficients (P1, P2), blunder point set to 5, computation of posterior standard deviation for images, high image location accuracy), in order to achieve corrected visually and geographically product with resolution of 5 mm on which we performed further GIS analyses, measurements, and calculations in ArcGIS Pro software ver. 3.2.2 (Esri 2024). All calculations and measurements were done on orthophoto mosaic product correspond to actual distances.

### Remote sensed data—drone images-based variables

Based on the created orthophoto mosaics we were able to determine vegetation (Prop_Vege) / water (Prop_Water) contribution cover in a 2-m buffer around the centre of nest bowl following marked as a point features^31^. In Prop_Vege, index we considered as vegetation aquatic plants including White water-lily, Yellow water-lily and Fringed water-lily, and also Water soldier, grass and dead vegetation present in BT nests. In Prop_Water index we included areas free of vegetation within created buffers. The information on proportion of area covered with open water and vegetation was derived from two-class rasters distinguished by a classification algorithm applied to orthophoto mosaic products (RGB multiband orthomosaic). For image classification we used the Support Vector Machine algorithm (SVM)—a commonly used machine learning approach dividing classes based on a provided final surface (here orthophoto mosaic) by maximizing border between classes of the dataset^42^. We used this classifier as it is less susceptible towards imperfections of provided image dataset (e.g., noise), which are very likely to happen in open environment with shifting weather and lightning conditions. SVM is also resistant to unbalanced number or size of training sites provided for each class^43^. Both types of cover (water and vegetation) were distinguished by SVM with maximal value set to 500 samples per class (Classify from classification tools, ArcGIS Pro software ver. 3.2.2 (Esri 2024)), based on manually picked training samples for both water and vegetation areas for each set of orthophoto mosaic pictures. We used from 100 to 300 samples (10 samples in each buffer on average), depending on the nest number in orthophoto mosaic and variability of light conditions within orthophoto mosaic. The selected range of samples provided us with the most satisfying results. We measured accuracy assessment of performed raster classification by the Stratified Random Sampling (hereafter SRS) method with 500 random points provided in the Accuracy Assessment tool in ArcGIS Pro software ver. 3.2.2 (Esri 2024). SRS was used to measure accuracy withing the created classes (vegetation, water)^44^. We used this assessment method since it considers the size of samples derived from each class. General values of accuracy of all created classified datasets were acceptable (Overall Accuracy: Kappa = 0.92 ± 0.07 for all studied colony cases combined). Finally, to obtain information on water/vegetation contribution ratio (Prop_Vege and Prop_Water) within a 2-m nest buffer, we summarized categorical raster dataset [Summarize categorical Raster tool, Image Analysis Tool, ArcGIS Pro software ver. 3.2.2 (Esri 2024)]. We were not able to obtain data on vegetation/water cover for *n* = 29; 9.63% of all detected nest sites in total, due to incomplete / insufficient image coverage of area. Such situations occurred when drone’s flight path became uneven, which was caused by wind disturbances (sudden gusts of wind). Such cases were excluded from the analyses.

### Remote sensed data—drone images-based analyses

From the created orthophoto mosaics we also obtained detailed data on nests location and distribution within the colony. To investigate inter- and intra-species differences between the colonies, we calculated several distance factors from applied point geometry features for all found nests. To find differences of colony structure we studied the shortest distances between neighbouring nests (hereafter Dist_Nests). To capture temporal growth of colony size we measured distances between nests and shoreline of the Druzno Lake (hereafter Dist_Shore) (longer distances from the shoreline suggest higher safety from land predators but also exposition to wind and waves in open lake areas) and between nests and geometric colony centre (centroid) (hereafter Dist_Centr) (indicator of colony growth / proportion). To identify general colony placement within area of the Druzno Lake we measured Dist_Shore mentioned earlier and distances between nests and Elbląg Canal (hereafter Dist_Canal) which is a potential disturbance factor (wave generator expressed by the distance from nests to the waterway). Elbląg Canal is a waterway mostly used nowadays by tourists (on board of ~ 15 m long touristic ships), anglers/fishermen and sailors, which use whether rowing boats, kayaks, speedboat or ships and yachts^45^ equipped with engines, activity of which generates waves and causes general disturbance to the birds nesting nearby. Distances in meters were calculated for all nests by the Near Tool in ArcGIS Pro software ver. 3.2.2 (Esri 2024). We distinguished the geographic centre of each colony (centroid) by the Mean Center Tool in ArcGIS Pro software ver. 3.2.2 (Esri 2024). To show how large area of the lake is covered by the colonies of the studied species, we calculated the potential area size of each surveyed colony by Minimal Convex Polygons (MCP) (e.g^46^). We generated and calculated MCP areas by the Minimum Bounding Geometry Tool (Data Management) by using convex hull on all point features (nests) belonging to a single colony in ArcGIS Pro software (Esri 2024) and reported them as means.

### Remote sensed data—satellite images-based variables

For assessing environmental conditions around nests (within a 7-m buffer), we used the Normalized Difference Vegetation Index (NDVI) as a proxy of vegetation density, and the Normalized Difference Water Index (NDWI) as a marker of open water contribution. NDVI values range from − 1 to 1, where < 0 = water, ~ 0 soil, 0.1–0.5 = sparse vegetation; >0.5 = dense vegetation. NDWI values range from − 1 to 1 which correspond to: -1 to -0.3 = absence of water / presence of vegetation; -0.3 to 0.0 = areas of moderate absence of water; 0.0 to 0.2 = humid areas; 0.2 < water areas.

To calculate these indices, we used daily images provided by Sentinel 2 mission [high resolution L2A tiff products (16-bit)] for June and July, acquired from browser.dataspace.copernicus.eu website, accessed on 30.08.2024). To achieve the most quality results, we used daily imagery with low to no cloud cover (≤ 35%)^47^. Due to cloudy weather during time of data collection, we were able to use one high-quality satellite image per month. We used the following bands: B3 (green, central wavelength: 0.560 nm, resolution 10 × 10 m), B4 (red, central wavelength: 0.665 nm, resolution 10 × 10 m), and B8A [near infrared (NIR) ‘Vegetation red edge’, 0.865 nm, resolution 20 × 20 m]^48^ for creating the following composite-derived indices: the Normalized Difference Vegetation Index (B4, B8A) and the Normalized Difference Water Index NDWI (B3, B8A). For both indices, we created two-layer composite rasters, from which we calculated indices (single band rasters of resolution 5.5 m), using following formulas: (B8A - B4) / (B8A + B4) for NDVI^32^ and (B3 − B8A)/(B3 + B8A) for NDWI^33^. We derived mean indices values from a histogram plot generated in ArcGIS Pro. Similarly, as^49^, we created a 7-m buffer zones around nests from which we derived information of environmental indices values (NDVI, NDWI) in proximity of nesting sites.

### Statistical analyses

All analyses were performed in R software ver. 4.2.2 (R Core Team 2022).

### Univariate analyses

We compared all measurements between the two species during the second control (04.06.2024) by the Wilcoxon test and for values regarding specific colonies of WT (*n* = 3) or for inter-colonial comparisons, we used the Kruskal-Wallis test. For the interspecific analysis, only data collected on the 2nd visit were considered due to sufficient sample size for both studied species. For the analyses regarding WT, we used data collected during the surveys from the 2nd to the 5th visit.

### Nesting site selectivity

To investigate preferences of both tern species towards some nesting traits we compared environmental indices values (NDVI, NDWI) within a 7-m buffer around existing nests and around 50 generated random points within not overgrown part of the Druzno Lake (the potential nesting range) covering 1,120.21 ha (Fig. 1). Similarly, we used this approach with two distance factors (Dist_Canal and Dist_Shore) to find any preferences for colony location in both species. Any significant differences between observed and randomly sampled values would indicate preferences towards habitats of specific characteristics or location and no differences would indicate no preferences. We performed this analysis on data collected during the second visit.

### Nesting niche width

We considered two types of nesting niches for the studied species based on selected environmental factors (Resource niches) and measured distances (Distance niches). Resource niche included remotely sensed indices (NDVI, NDWI) from 7-m buffers around nests. Distance nesting niche is defined by values distances measured between nests and Elbląg Canal (Dist_Canal), between nests themselves (Dist_Nest), and between nests and shoreline (Dist_Shore) abundantly covered with reeds. We compared distances between nests and shoreline of the Druzno Lake to assess colony location within the lake. We also compared measured distances between nests and colony centroid, nests and Elbląg Canal, and between nests themselves to describe the structure of tern’s colonies to find any interspecific differences. For quantifying n-dimensional ecological niches and niche overlap in two studied tern species we used the *nicheROVER* package^50,51^. With use of the Bayesian Inference Framework, we depicted niches as regions of 95% probability and at 1,000 runs (for higher results accuracy). To visualize niche characteristics for considered environmental components, we plotted 2-dimensional niche projections. Finally, we calculated the size of nesting niches, which enabled us to compare their mutual overlap^51^. We computed interspecific niche overlap by Monte Carlo calculation of niche region overlap metrics. For this, we set two values for the scalar of niche region sizes (alpha = 95 and alpha = 99). We calculated niche overlap in the *nicheROVER* package^50,51^. We compared niche width between the studied tern species using Wilcoxon test in the *rstatix* package (ver. 0.7.2)^52^. We also compared the width of nesting niches for WT from the 2nd to the 5th visit to find any changes occurring with progress of breeding season. We used the Wilcoxon test (for the 2nd visit on 04.06, when we observed two colonies) and Kruskal-Wallis test for the rest of visits.

### Spatial niches

To describe nesting niches of both species in space, we determined species composition (mixed or uni-species) in all found colonies. Finally, we compared interspecific distances between centroids of all detected colonies within area of the Druzno Lake.

### Inter-species differences in nesting sites

To compare nest characteristics between species, we used all considered resource variables (NDVI, NDWI, Prop_Water, Prop_Vege) and measured distance factors (Dist_Canal, Dist_Centr, Dist_Nest, Dist_Shore) and applied them in Conditional Inference Trees (CIT). CIT belongs to a non-parametric class of regression trees, that apply tree-like regression models to possible inference theories^53^. With this method, we were able to determine significant differences in the frequency of occurrence for species or WT colonies under certain values of factors. We created CIT with the following formula: SPECIES ~ NDVI + NDWI + Prop_Water + Prop_Vege + Dist_Canal + Dist_Centr + Dist_Nest + Dist_Shore. We performed CIT analyses in the *partykit* package^53^.

### Spatio-temporal niches

To investigate changes occurring nesting niches in time (advancement of the breeding season) for WTs, we performed CIT analyses in the *partykit* package^53^ with the following formula: COLONY ~ Visit_number + NDVI + NDWI + Prop_Water + Prop_Vege + Dist_Canal + Dist_Centr + Dist_Nest + Dist_Shore. We did not perform this analyse for BT, due to presence of this species only during one visit. We also computed n-dimensional ecological niches for all colonies using the *nicheROVER* package^50,51^.

## Results

During all aerial (drone) controls (covering a total area of 32.5 ha) we detected two BT (hereafter BT_A; BT_B) and three WT (WT_A; WT_B; WT_C) colonies in Druzno Lake (Fig. 1). All detected colonies were uni-species. BT colonies accommodated 24 breeding pairs (Table S1_1 in Supplementary Materials) and covered generally smaller area (mean MCP area: 0.97 ha) compared to WT colonies (mean MCP area: 1.61 ha) accommodating from 45 to 98 nests (Table S1_1 in Supplementary Materials), but the difference in area covered was not significant (Wilcoxon test; W = 9, *P* = 0.77; df = 2, 13). All BT and WT colonies covered 0.17% and 0.59% of area of Druzno Lake suitable for marsh terns breeding (open water zone and area covered with floating vegetation, 1,120.21 ha).

### Nest site characteristics

**Fig. 3:**
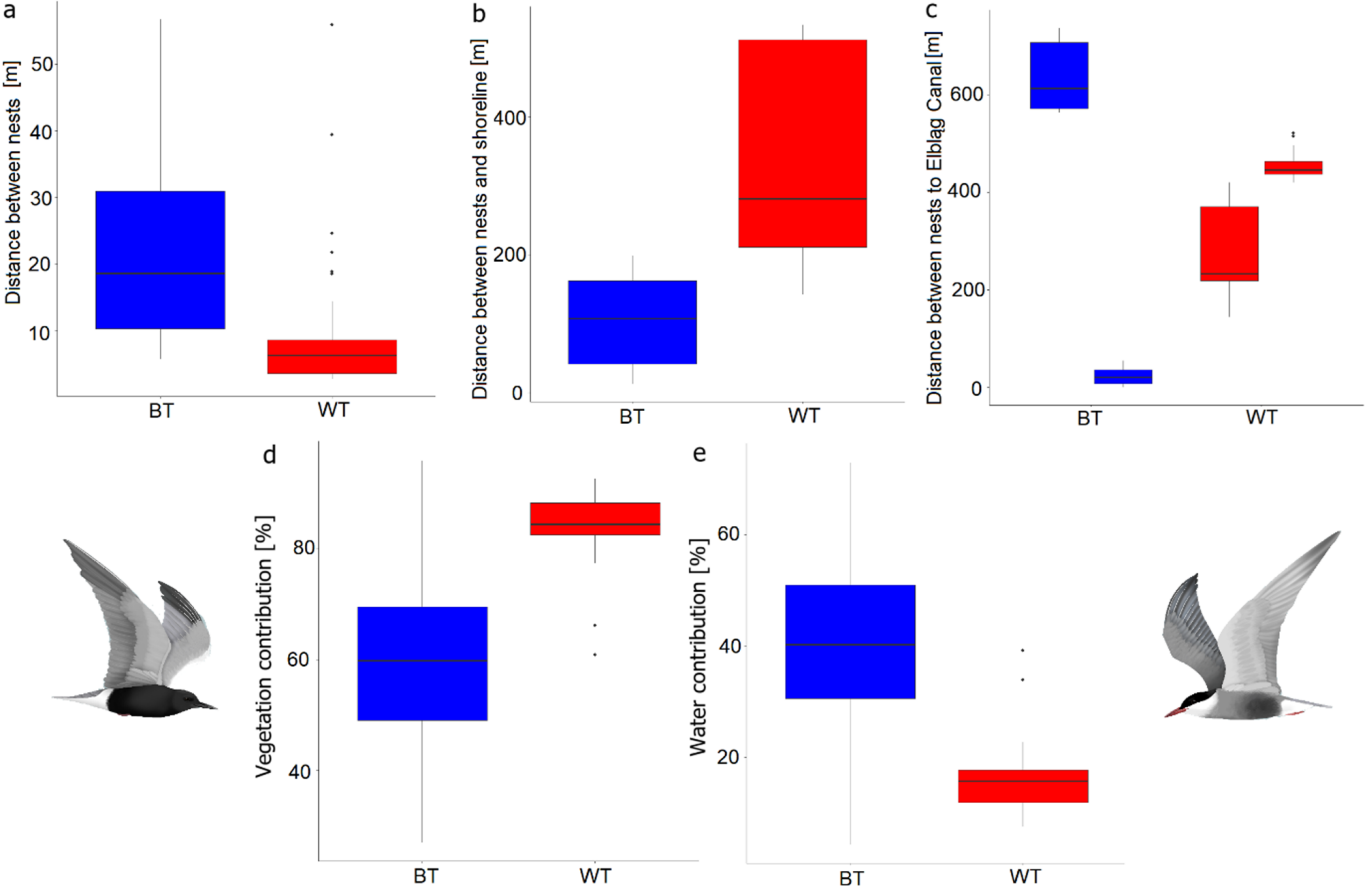
Inter-species differences in distances, between nests within colonies (**a**), nests and shoreline (**b**), inter-colonial distances between nests and the Elbląg Canal (**c**), vegetation area contribution (**d**) and water area contribution (**e**) within a 2-m radius buffer around nesting site of BT – Black Tern, WT – Whiskered Tern. Boxplots show the median (band inside the box), the first (25%) and third (75%) quartile (box), the lowest and the highest values within 1.5 interquartile range (whiskers), and outliers (dots). Graphics of terns by K.C.

In WT colonies nests were located closer to each other compared to BT (Wilcoxon test; W = 897; *P* < 0.001; *n* = 45) (Fig. 3a). WT also located nests further from the lake shore, compared to BT (Wilcoxon test; W = 23; *P* < 0.001; df = 2, 69) (Fig. 3b; Table 1). Distances between nests to colony centre (centroid) were similar in both species (Wilcoxon test; W = 568; *P* = 0.73; df = 2, 69). We also detected significant inter-colonial differences in proximity to the Elbląg Canal between both species (Kruskal-Wallis test: χ^2^_3_ = 62.441, *P* < 0.001) (Fig. 3c; Table 1) (see details about distances between nests and Elbląg Canal, shoreline of the Druzno Lake, nests, and centroid of colony for particular WT colonies in Tables from S1_2 to S1_5 and figures from S1_1 to S1_4 with test results Table S1_6 in Supplementary Materials S1).

We found that WTs were breeding in areas with significantly higher vegetation contribution in close proximity of nesting sites (Wilcoxon test; W = 66; *P* < 0.001; df = 2, 62) and significantly lower water contribution (Wilcoxon test; W = 992; *P* < 0.001; df = 2, 62) compared to BT (Fig. 3d, e).

**Table 1:** Median (Interquartile range) of all measured distances between black Tern and whiskered Tern nests and selected features during the 2nd visit (04.06.2024). Significant interspecies differences are bolded.

Distance [m] between the nest and	Black Terns	Whiskered Terns
Elbląg Canal	39.69 (563.53)	419.57 (212.96)
centroid of the colony	66.74 (27.43)	66.29 (53.56)
the nearest nest	**18.66 (20.65)**	**6.31 (5.05)**
shoreline of the Druzno Lake	**107.75 (120.88)**	**281.49 (01.01)**

During the 2nd visit (04.06.2024) Dist_Canal, Dist_Centr, and Dist_Shore differed significantly between two colonies of WT. During the 5th visit (19.07), all colony measurements differed significantly between three present colonies. On 26.06 and 08.07, only Dist_Nest and Dist_Canal differed significantly between colonies of this species of terns.

At WT_A colony, distances between nests and Elbląg Canal decreased with progress of the breeding season (Kruskal-Wallis test; Chi^2^ = 16.184; df = 3; *P* = 0.001), while distances between nests and colony centroid increased (Kruskal-Wallis test; Chi^2^ = 38.355; df = 3; *P* < 0.001). We found no differences in distances between nests (Kruskal-Wallis test; Chi^2^ = 0.484; df = 3; *P* = 0.92) and between nests and shoreline (Kruskal-Wallis test; Chi^2^ = 1.2773; df = 3; *P* = 0.73).

At WT_B Colony distance between nests and shoreline shortened with time (Kruskal-Wallis test; Chi^2^ = 46.131; df = 3; *P* < 0.001). The rest of traits increased significantly with progress of the breeding season (Kruskal-Wallis test, Chi^2^ = 46.176; df = 3; *P* < 0.001 for Dist_Canal, df = 3; Chi^2^ = 39.334; *P* < 0.001 for Dist_Centr, Chi^2^ = 7.965; df = 3; *P* < 0.05 for Dist_Nest).

At WT_C Colony with progress of the breeding season, distance between its centroid and nests increased significantly (Kruskal-Wallis test; Chi^2^ = 6.9226; df = 2; *P* = 0.03), as well as the distance between nests and shoreline increased significantly (Kruskal-Wallis test; Chi^2^ = 14.596; df = 2; *P* = 0.0007). Distance between nests and the waterway decreased significantly with progress of the breeding season (Kruskal-Wallis; Chi^2^ = 17.023; df = 2; *P* = 0.0002). Distances between nests were similar during all controls (Kruskal-Wallis; Chi^2^ = 0.436; df = 2; *P* = 0.80).

### Nesting sites selectivity

**Fig. 4:**
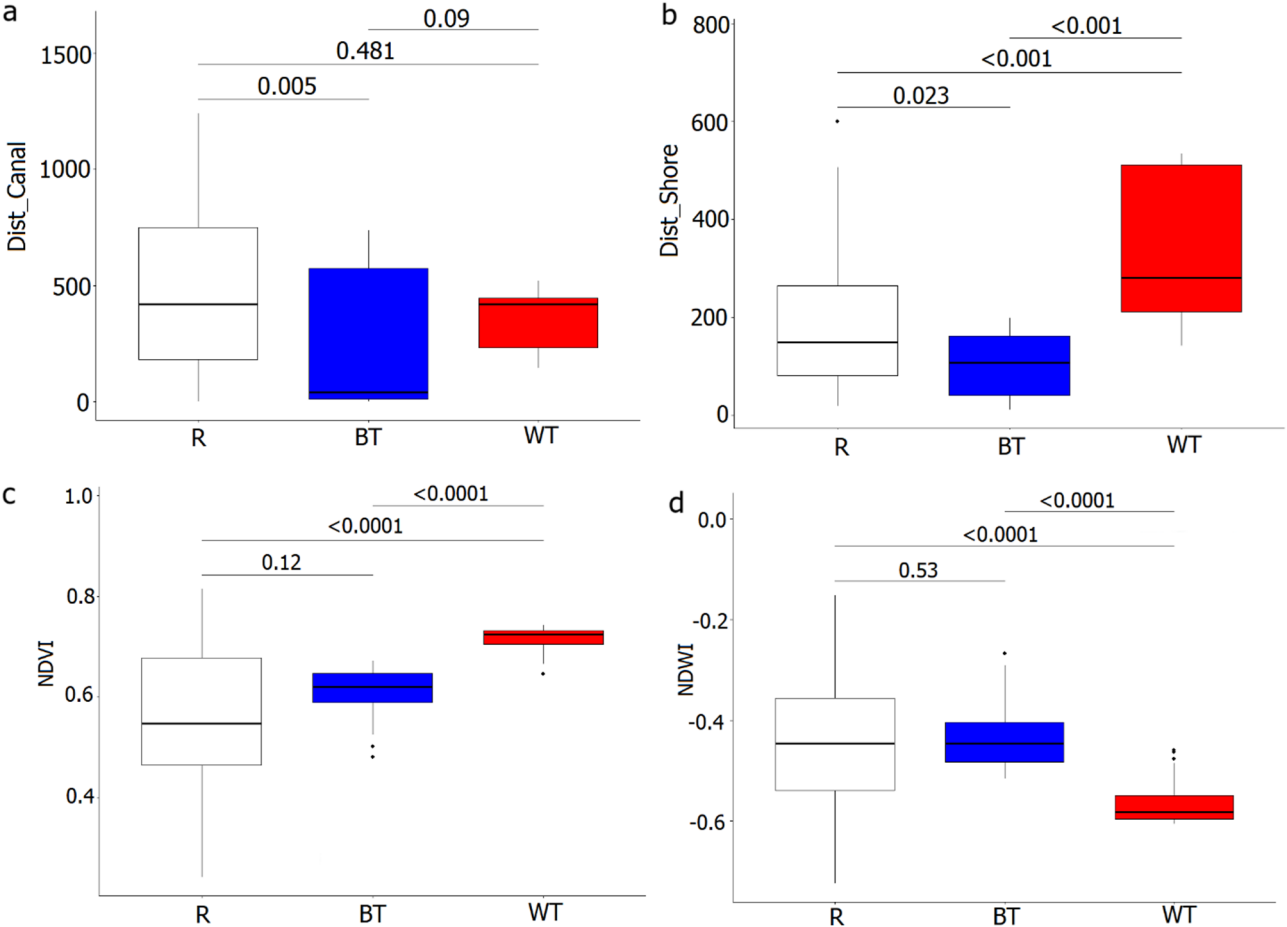
Comparison of distances between nests and Elbląg Canal (Dist_Canal) (**a**), distances between nests and shoreline of the Druzno Lake (Dist_Shore) (**b**), Normalized Difference Vegetation Index NDVI (proxy of vegetation density) (**c**), and Normalized Difference Water Index NDWI (proxy of open water contribution) (**d**). Mean values between Black Terns (BT), Whiskered Terns (WT) and random points in Druzno Lake (R). Boxplots show the median (band inside the box), the first (25%) and third (75%) quartile (box), the lowest and the highest values within 1.5 interquartile range (whiskers), and outliers (dots). Horizontal lines above boxplots show P values of Wilcoxon test for values for inter-group comparisons.

When comparing values of two measured factors (Dist_Canal, Dist_Shore) between real nests and random points, we found that BTs preferred nesting closer to the Elbląg Canal (Wilcoxon test; W = 841.5; *P* = 0.005; df = 2, 74), but WTs had no significant preference for this feature (Wilcoxon test; W = 1220; *P* = 0.48; df = 2, 95). Also, we found no inter-species differences in distance to the canal (Wilcoxon test; W = 675; *P* = 0.09; df = 2, 95) (Fig. 4a). We found that both species exhibit preferences for forming colonies at specific distances from the lake shoreline. BT preferred nesting closer to the shoreline, while WT further from the lake shore (Wilcoxon test; W = 797; *P* = 0.02; df = 2, 74 W = 475, *P* < 0.001; df = 2, 95 for BT and WT, respectively) (Fig. 4b). The inter-species difference in distance, from the nest to the nearest lake shore was significant (Wilcoxon test; W = 1057; *P* < 0.001; df = 2, 69) (Fig. 4b). We recorded significant interspecific differences in environmental indices in nests neighbourhood (Wilcoxon test; W = 22; *P* < 0.001; df = 2, 62 for NDVI and W = 1186; *P* < 0.001; df = 2, 62 for NDWI, respectively), where BT nests were characterized by both lower NDVI (reflecting lower vegetation density) and higher NDWI (reflecting higher contribution of open water areas), while WT the opposite (Fig. 4c, d). We found no significant differences in open water contribution and vegetation density (NDWI and NDVI values) between real nests and random points for BTs (Wilcoxon test; W = 464; *P* = 0.12; df = 2, 72 and W = 545; *P* = 0.53; df = 2, 72 for NDVI and NDWI, respectively) (Fig. 4c, d). In contrast, WTs preferred higher values of NDVI (reflecting higher vegetation density) and lower values of NDWI (reflecting smaller open areas contribution) (Wilcoxon test; W = 382; *P* < 0.001; df = 2, 90 and W = 2000; *P* < 0.001; df = 2, 90 for NDVI and NDWI, respectively) compared to random points (Fig. 4c, d).

**Fig. 5:**
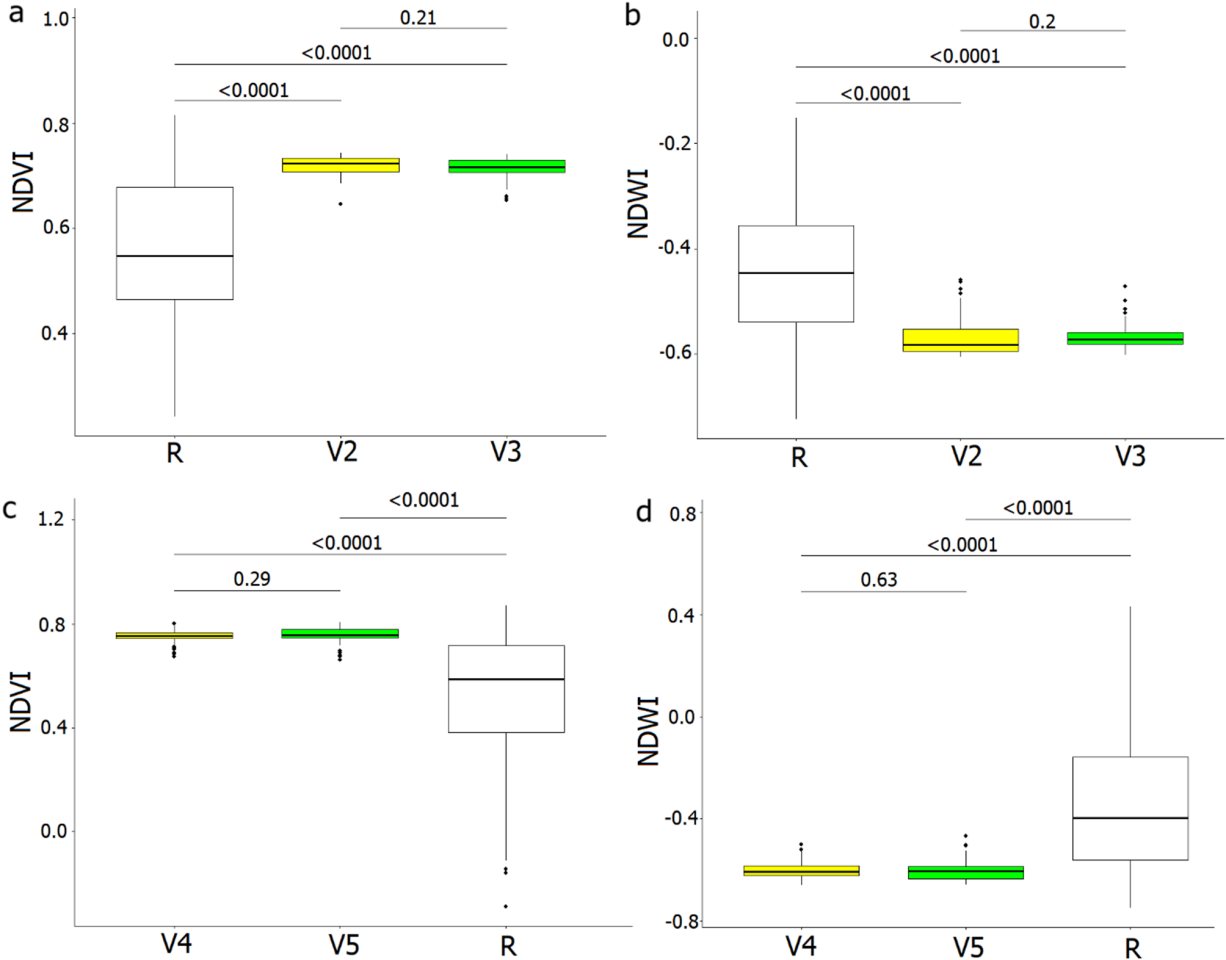
Normalized Difference Vegetation Index NDVI (proxy of vegetation density) (**a**,**c**) and Normalized Difference Water Index NDWI (proxy of open water contribution) (**b**,**d**) in observed Whiskered Terns colonies and in potential nesting sites (random points) during the following visits: V2–2nd visit (04.06.2024); V3–3rd visit (26.06.2024); V4–4th visit (08.07.2024); V5–5th visit (19.07.2024). Visits grouped to V2 and V3, and V4 and V5 by the availability of NDVI and NDWI data. Boxplots show the median (band inside the box), the first (25%) and third (75%) quartile (box), the lowest and the highest values within 1.5 interquartile range (whiskers), and outliers (dots). Horizontal lines above boxplots show P values of Wilcoxon test for inter-group comparisons.

We found significant differences for both environmental indices (NDVI, NDWI) between random points and nest location in every WT colony recorded between 04.06 and 19.07, when birds preferred higher NDVI values (reflecting denser vegetation) and lower NDWI values (reflecting smaller open areas contribution) (Kruskal Wallis test; *P* < 0.001; df = 3 for all four visits). NDVI and NDWI values from the same month at nesting sites were similar during the consequent visits (Wilcoxon test; *P* > 0.05; df = 2, 130 for the 2nd and 3rd visits and Wilcoxon test; *P* > 0.05; df = 2, 178 for the 4th and 5th visits) (Fig. 5).

### Factors affecting nesting sites characteristics

**Fig. 6:**
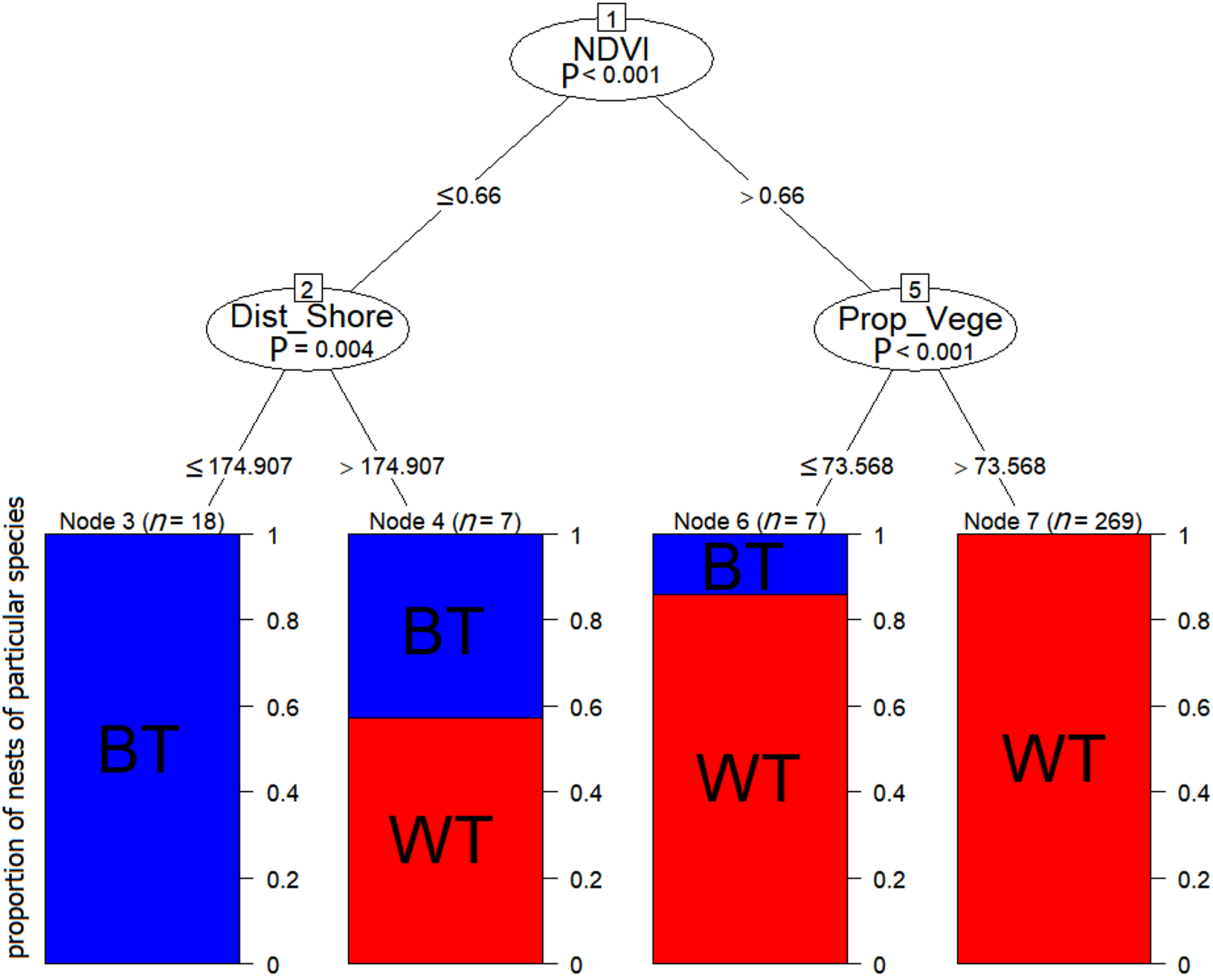
Conditional inference tree (CIT) characterizing statistically significant factors affecting the distribution patterns of nests within colonies of Black Tern (BT) and Whiskered Tern (WT). The following factorial predictors were used as initial predictors: NDVI (proxy of vegetation density), distances between nests and shoreline of the Druzno Lake (Dist_Shore), and contribution of vegetation cover within a 2-m radius from the nest (Prop_Vege). Encircled variables have the strongest association with the response variable (species). The P values listed at each encircled node represent the test of independence between the listed variable set and the response variable. Terminal nodes (leaves) indicate variable levels characterizing the response variable and n indicates the number of nest cases. Histograms in leaves indicate the frequency of particular species nest case: blue – BT and red – WT.

CIT indicated that among all considered components (both distance and habitat) some distance (Dist_Shore) and habitats components (Prop_Water, NDVI) had statistically significant effect on nests characteristics of both species (Fig. 6). It distinguished four clusters (terminal nodes) – two uni-species and two mixed species characterized by different environmental characteristics (Fig. 6). The first split divided observed nests into groups with various NDVI values: Node 2 (≤ 0.66) and Node 5 (> 0.66). Both nodes were further split but by different components. Node 2 was separated into two groups by measured Dist_Shore lower than 174.907 m (Node 3) and more than 174.907 m (Node 4). Node 3 was characterized by 100% contribution of BT, while in the next node (Node 4) by the similar contribution of WT (57.1%) and BT (42.9%). Node 5 was further separated by Prop_Vege value: Node 6 (≤ 73.568) and Node 7 (> 73.568). Node 6 with lower contribution of floating vegetation around nest was characterized by the prevalence of WT (85.7%) and lower contribution of BT (14.3%). The last node with higher contribution of floating vegetation around nest was characterized by 100% contribution of WT (Fig. 6).

### Spatial niches

Both species were breeding in separate parts of Druzno Lake (Fig. 1)—they did not form mixed colonies. Mean (± SD) distances between centroids of colonies of both species was 2,672.75 (± 1,879.18) m and ranged from 588.63 to 5,332.13 m.

### Nesting niches

**Fig. 7:**
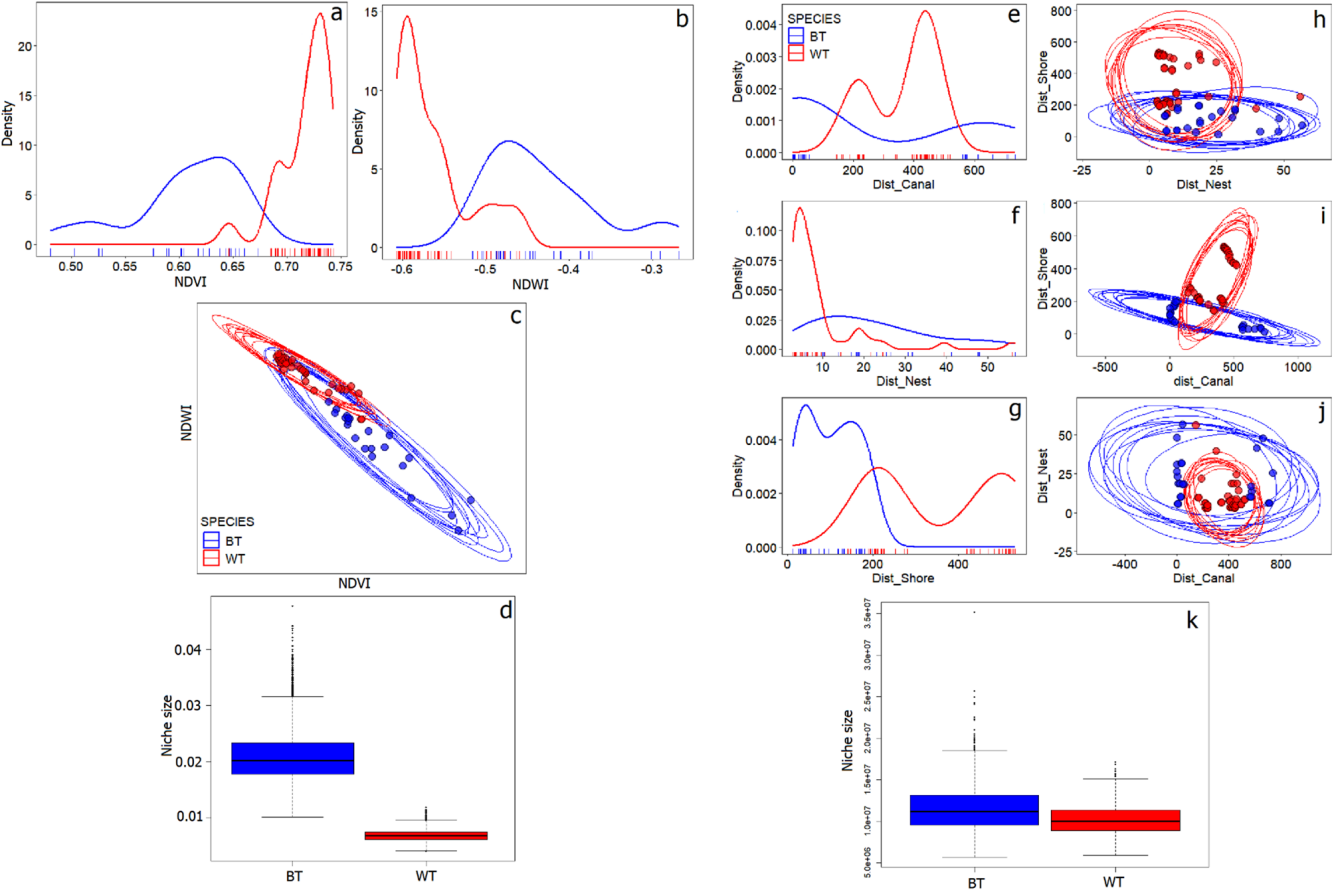
Visual projections of resource (**a**–**d**) and distance (**e**–**k**) nesting niches of two marsh tern species: BT – Black Tern and WT – Whiskered Tern. (**a, b,e, f,g**) – distribution of particular environmental variables displayed as one‑dimensional density plots; (**c**,**h**,**i**,**j**)—ellipses overlapped with two-dimensional scatterplots, representing (*n* = 10) randomly sampled projections of the nesting niches in two‑dimensional perspectives defined by two environmental variables. Nesting niche size comparison (**d**,**k**). Boxplots in (**d**,**k**) plots show the median (band inside the box), the first (25%) and third (75%) quartile (box), the lowest and the highest values within 1.5 interquartile range (whiskers), and outliers (dots).

We found that both resource [niche size (EST ± SE): 0.021 ± 0.004 for BT and 0.007 ± 0.001 for WT, and distance nesting niches [niche size (EST ± SE): 11,631,312 ± 3,028,423 for BT, and 10,249,507 ± 1,921,648 for WT] were significantly wider for BT than WT (Wilcoxon test; V = 2,001,000; *P* < 0.001; df = 2, 69, for both niche types) (Fig. 7). We found that resource nesting niche of WT was overlapping (95% overlap) with BT by nearly 50%. Distance nesting niches of both tern species overlap moderately (~ 25%) (Table 2).

We found that resource niches for both tern species did not overlap considerably in NDVI values, but did overlap for NDWI values at ~-0.53 value. Distribution of NDVI values for nesting sites of BT exhibited two peaks (smaller at ~ 0.5 and bigger at ~ 0.65) and for NDWI (at ~-0.5 and at ~-0.3). In contrast, WT was characterized by several peaks with higher NDVI values (smaller ones at ~ 0.65, 0.68, and the biggest at 0.72), and for NDWI it showed two peaks (larger at ~ -0.6; and smaller at ~-0.5) (Fig. 7).

Distribution of Dist_Canal values for both species exhibited two peaks, but they do not overlap with peaks for BT at ~ 0 m and 750 m, and for WT at 200 m and 450 m. Distribution of Dist_Nest for BT showed a single peak for values ranging from 10 to 20 m, while WT exhibited high peak at ~ 5 m an several smaller ones at ~ 20 m, and ~ 40 m. Values for Dist_Shore showed distinct density pattern for both species with two peaks at 5 m and ~ 180 m for BT and at ~ 200 m and ~ 500 m for WT.

**Table 2:** Report of 99% and 95% overlap between mean values of two types of niches in BT – Black tern and WT – Whiskered tern.

Species	Overlap	Black Tern	Whiskered Tern
Black Tern	99% resource niche	-	10.86
Whiskered Tern	99% resource niche	81.94	-
Black Tern	95% resource niche	-	7.22
Whiskered Tern	95% resource niche	48.62	-
Black Tern	99% distance niche	-	37.04
Whiskered Tern	99% distance niche	13.51	-
Black Tern	95% distance niche	-	24.17
Whiskered Tern	95% distance niche	10.43	-

### Spatio-temporal niches

We found that both types of niches in WT were similar between colonies (Kruskal-Wallis test; df = 2; *P* > 0.05) and consecutive visits (Kruskal-Wallis test; df = 3; *P* > 0.05). However, resource niches differed significantly between colonies (Kruskal-Wallis test; df = 2; *P* < 0.05). The widest resource niche characterized WT_A colony, followed by WT_B, while the smallest one described WT_C colony (Fig. S1_5 and Table S1_7 in Supplementary Materials S1).

**Fig. 8:**
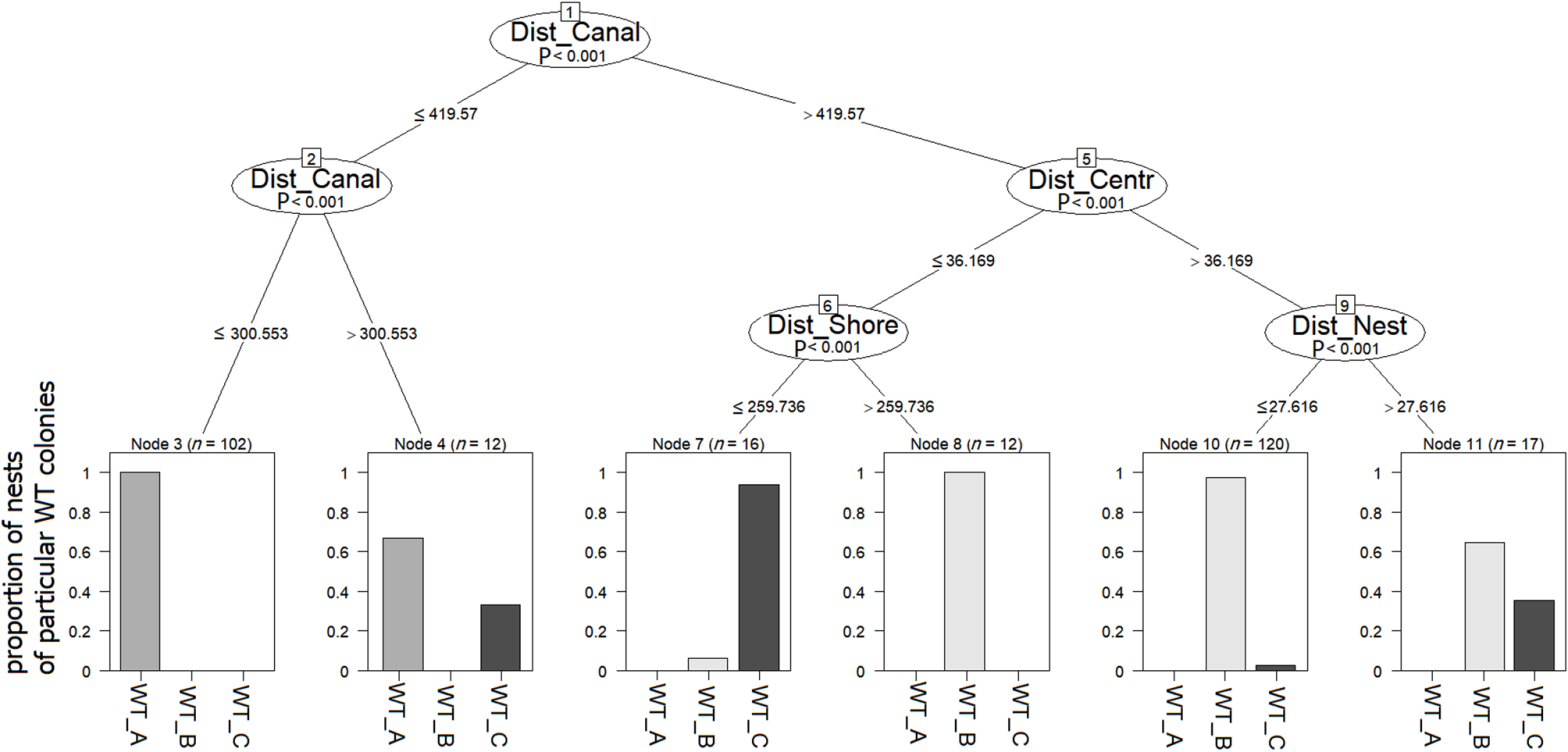
Conditional inference tree (CIT) characterizing statistically significant factors affecting the distribution patterns of nests of Whiskered Terns in particular colonies. Encircled variables have the strongest association with the response variable (colony). The P values listed at each encircled node represent the test of independence between the listed variable set and the response variable. Terminal nodes indicate variable levels characterizing the response variable and n indicates the number of nest cases. Histograms in terminal nodes indicate the frequency of nests in particular colonies (WT_A, WT_B, WT_C) with given characteristics. Dist_Canal – distances between nests and the Elbląg Canal, Dist_Centr – distances between nests and centroid of the colony, Dist_Nest – distances between nests within single colony, Dist_Shore – distances between nests and Druzno Lake shoreline.

CIT indicated that among all considered components (Visit_number, NDVI, NDWI, Prop_Water, Prop_Vege, Dist_Canal, Dist_Centr, Dist_Nest, Dist_Shore) - only some distance components (Dist_Canal, Dist_Centr, Dist_Nest, Dist_Shore) had statistically significant effect on nests features in particular WT colonies (Fig. 8). It distinguished six clusters (terminal nodes) with different nests characteristics (Fig. 8). The first split divided observed nests into groups differing in Dist_Shore: Node 2 with distance < 419.57 m and Node 5 > 419.57 m. Node 2 was further separated into two groups again by Dist_Shore with values ≤ 300.55 m characterizing Node 3 and > 300.55 m Node 4. Node 3 was characterised by a 100% contribution of WT_A colony, while Node 4 was characterized by a prevalence of WT_A colony (66.7%) and lower contribution of WT_C (33.3%). Node 5 was further split by Dist_Centr, ≤ 36.17 (Node 6) and > 36.17 (Node 9). Node 6 was further split by Dist_Shore into two final groups, ≤ 259.74 (Node 7) and > 259.74 (Node 8). Node 7 was characterized by a 6.2% contribution of WT_C and 93.8% contribution of WT_B colony. Node 8 was characterized by 100% contribution of WT_B colony. Node 9 further split into two final groups by Dist_Nest ≤ 27.62 (Node 10) and > 27.62 (Node 10). Node 10 was characterized with a 97.5% contribution of WT_B and 2.5% WT_C. Node 11 was described by 64.7% prevalence of WT_B and 35.3% contribution of WT_C colony (Fig. 8).

## Discussion

In this study combining aerial and satellite remote sensing data we found niche partitioning in space and nesting habitat between Black Terns (BT) and Whiskered Terns (WT) breeding sympatrically in the hypertrophic lake. In contrast to results from the same site from the 2000s^20^ and some reports from other breeding sites^19,21^ the studied species did not form mixed colonies. Presumably in the past, colonies of BT may have attracted new coming WTs. Spatial isolation of breeding sites in those species observed in this study can be explained by partitioning of used resources to reduce the negative impact of the interspecific competition. Distances between nests in BT colonies were similar to ones reported in literature ranging from 1 to 19 m^18,20^. Similarly, values for WT (1.59–62.73 m; mean = 11.24) were similar to the ones reported for the whole range (ranging between 1 and 50 m with the most common ones 1–5 m^18^), and for the same breeding site in the 2000s (ranging between 3 and 130 m^20^).

We found that BT showed big variance in distances to waterway, the Elbląg Canal, as one colony was formed in the middle of this waterway, and the second far away from it, close to the lake shoreline. WT generally formed colonies at intermediate distances (144.30–795.25 m) from this canal. BT generally formed colonies closer to the shoreline, with nests were placed further away from the nearest neighbours, in contrast to WT which did the opposite. Location of BTs nests near wall of reedbeds of the waterbody also reported by^30^ was probably attributed to shielding from the wind^20^, unfavourable weather conditions^30,55^, or/and reduce nest detectability by avian predators. Unfortunately, during this study, vegetation cover failed to protect nests from fluctuating water level and exposure to waves and disturbances from the fleet activity. WT formed colonies in open areas of the lake as reported earlier^20^. Colony formation in this species appears to be not limited by nest architecture, as vegetation platforms seem to be more durable and resistant to wind. Lush vegetation cover also provides waves attenuation (e.g.^56^), resulting in lower nest disturbances. For this, WT appears to be selective towards areas on the lake with abundant areas of floating vegetation.

We found that BT, in contrast to WT, preferred nesting habitats with greater contribution of water in the neighbourhood and sparse vegetation cover. Our results on characteristics of nesting habitat in BT are in concordance with literature showing, i.e., relatively high water contribution near nesting site (e.g.^34,57,58^), building nests in the proximity of helophytes^30^ (in this research—shoreline), avoidance of open water zone^20,54^. Our results on WT’s nesting habitat selection are also similar to the ones reported in literature, indicating preference to nesting in water zones located far from the shoreline^20^, with extensive cover of nympheids (low contribution of open water areas)^35^. Different preferences for vegetation types can explain observed interspecific spatial partitioning (selection of microhabitats). Preferences towards different type of vegetation could have also resulted from different arrival time on the breeding grounds in both studied species. Based on observation of flying juveniles in monitored colonies (for details, see Supplementary Materials S1), we estimated that BT arrive ~ 3 weeks earlier than WT in the studied year. Vegetation at stage of BT arrival at Druzno Lake, i.e., beginning of May^22^ is insufficient or not well developed (authors’ observations) for being utilized as a foundation substrate of tern’s nest, making them select locations which other important traits like proximity to reedbeds or shoreline securing protection against winds and waves. WTs which arrive about month later can select sites with well-developed floating vegetation. Thus, in the studied year, we were able to detect and confirm interspecific spatial partitioning of nesting niche between both tern species. Observed interspecific differences in selected breeding habitat, might suggest that decline of BT could be related to a wider range of environmental factors including prey availability or water level fluctuations.

In contrast to our expectation, BT had wider nesting niches than WT, which suggests that the former species is capable of utilising bigger variety of potential nesting habitats. BT was also not selective towards habitats with lower vegetation density (NDVI) and open water area contribution (NDWI). WT, on the other hand, can be described as a specialist due to species habitat selectivity. WT seem to select habitats abundant in floating vegetation, especially White water-lily beds. As we expected, we found that nesting niche size of WT increased with the progress of the breeding season, indicating use of diversified nesting habitats. It can be explained by the fact that late breeders were probably forced to select suboptimal nesting sites. Those would include locations vulnerable to predation^59^, located in peripheral zone of the colony, e.g,^60^, and/or places with higher water contribution (more energy is used to build a vegetation platform).

One important difference between both study species is architecture of built nests. WTs form durable, vegetation platforms on big nympheid species, i.e., Yellow water-lily, White water-lily, or Fringed water-lily^22,35,61^. Such placement on water-lily beds protects nests from water level fluctuations and prevents drifting. We observed (as reported before (e.g^20,22,62^), that BT formed small nests on dead vegetation, mud mounds, or muddy and grassy movable islets often with water soldiers (usually several cm above water level). Such structures are not resistant towards alternating water levels and are susceptible to drowning or being swept away by wind and waves (authors’ observations).

In last decades, decline of BT populations are observed worldwide, including Europe (e.g.^26,63^). and Northern America (e.g.^64,65^). The reason behind this decline is not clearly recognized, but it is possibly caused by combination of various factors, which include, e.g., unfavourable weather conditions (strong wind and rain), waves, poor vegetation protection^66^, but also climatic changes and anthropogenic habitat influences^64^. Frequent events of collapsing BT colonies may lead to intense loss of number of active breeders, resulting in already observed poor genetic diversity of BT population^67^, endangering species for local extinctions. Possible actions to limit breeding losses may include installation of artificial nest platforms. They were found as an effective management tool to enhance nesting habitat for BTs in the United States^68^ and the Netherlands^69^. It has been found that BT prefer broader platforms, which presumably are less susceptible to waves and changes in water level^70^. Nevertheless, nesting on breeding platform might bring threat to its user due to high visibility and thus easier detectability by avian predators^69^. For this species using anchored artificial platforms of size 40 × 50 cm, would be recommended as suggested by^69^. Such platforms should be well hidden in vegetation, camouflaged (base covered with different nesting material) and have flat edges to allow easy escape for chicks in danger. The use of such platforms also needs to be temporarily controlled, e.g. platforms should be placed at an appropriate time during the breeding season of BTs to avoid occupation of the platform by a competing species breeding earlier, e.g. Black-headed Gull (*Chroicocephalus ridibundus*) (authors’ observations). Our results provide insights into the nesting habitat requirements of this declining marsh tern species, with implications for conservation planning. Annual aerial controls to investigate location and size of colonies in relation to condition of habitat could also be applied as a conservation measure of BT, as it was conducted successfully throughout this study. Use of artificial nesting platforms, regular population controls, and imposition of regulations on fleet activity in near proximity to the colony sites during the breeding season, could improve breeding success and population condition of this species.

### Limitations of the study

We are aware of some limitations of this study. First, we performed the study in one year with specific fluctuations of water level. Second, we collected data for BTs only during a single visit, while for the WTs during all visits. Yet loss of the BT colonies was dictated by stochastic events (e.g., sudden increase of water level and intensive fleet activity on the Elbląg Canal). Third, we did not analyze the temporal aspect of niche partitioning between species due to too small number of visits with both species present. We also did not study vegetation/water coverage in all the studied nesting sites due to lack of sufficient orthophoto mosaic cover. Finally, we also were not able to obtain information on NDVI and NDWI for every visit due to dense cloud cover over study area; thus, we used the same daily raster per month. Nevertheless, our results still provide valuable insights into nesting niches of two marsh tern species.

## Conclusions

This is one of the first comprehensive studies of nesting habitat niches partitioning between two sympatrically breeding marsh tern species. We found that both studied species, besides several similarities (e.g., main prey items or nesting habitat), exhibit some differences in nesting sites characteristics. Spatial partitioning is expressed by formation of uni-species colonies in different parts of the lake. Colonies of both species differed in environmental properties (contribution of water/vegetation areas or vegetation density) in the neighbourhood. Nesting niches of BT were larger compared to WT being selective towards some environmental conditions within nesting area. This study indicates that a hypertrophic lake may facilitate sympatric breeding of two closely related species thanks to spatial and nesting habitat niche partitioning. Interspecific differences in nest architecture and colony location (distances to the lakeshore and human disturbances areas) may result in different breeding output and presumably could be responsible for the observed opposite demography trends in both species in the study area.

## Electronic supplementary material

Below is the link to the electronic supplementary material.

Supplementary Material 1

## Data Availability

The datasets created and analysed this study are available from the corresponding author at a reasonable request.
