# Supplementary material for: Nesting niche partitioning between two sympatrically breeding Chlidonias Tern species revealed by remote sensing
Source: Sci Rep. 2025 Jul 1;15:21701. doi: 10.1038/s41598-025-06205-4 (PMC12216925; doi:10.1038/s41598-025-06205-4)
Supplement: Supplementary file 1 — Supplementary Material 1 [file 41598_2025_6205_MOESM1_ESM.pdf]

Nesting niche partitioning between two sympatrically breeding *Chlidonias* tern species revealed by remote sensing

Karolina Cieślińska★, Brygida Manikowska-Ślepowrońska, Krzysztof Ślepowroński, Dariusz Jakubas

Department of Vertebrate Ecology and Zoology, Faculty of Biology, University of Gdańsk, Wita Stwosza 59, Gdańsk 80-308, Poland

★Corresponding author

E-mail: karolina.cieslinska7@gmail.com

Supplementary Materials S1 Reports on number of breeding pairs in both species, number of nests with eggs, number of nests with hatchlings; compilation of measured distances in WT colonies; niche sizes and their projections for WT colonies in consecutive field visits.

Table S1_1. Summary of the number of observed breeding pairs (based on counted nests from aerial survey), nests with minimum single egg, nests with at least one chick: of Black Tern (BT) and Whiskered Tern (WT) breeding in the Druzno Lake in N Poland during the consequent visits in 2024. * – observed pair without nest found; ** – date of observation of first flying fledglings of BT; *** – date of observation of first flying fledglings of WT, MCP – Minimum Convex Polygon.

<table>
<tr><th rowspan="3">Date (Number) of visit</th><th colspan="6">Number of</th><th colspan="2" rowspan="2">Mean Estimated Colony size (MCP) per visit [ha]</th></tr>
<tr><th colspan="2">Breeding pairs</th><th colspan="2">Nests with eggs</th><th colspan="2">Nests with hatchlings</th></tr>
<tr><th>BT</th><th>WT</th><th>BT</th><th>WT</th><th>BT</th><th>WT</th><th>BT</th><th>WT</th></tr>
<tr><td>18.05.2024 (1st)</td><td>1*</td><td>0</td><td>0</td><td>0</td><td>0</td><td>0</td><td>0</td><td>0</td></tr>
<tr><td>04.06.2024 (2nd)</td><td>24</td><td>45</td><td>4</td><td>8</td><td>0</td><td>0</td><td>0.97</td><td>1.29</td></tr>
<tr><td>26.06.2024 (3rd) **</td><td>0</td><td>85</td><td>0</td><td>13</td><td>0</td><td>1</td><td>0</td><td>1.70</td></tr>
<tr><td>08.07.2024 (4th)</td><td>0</td><td>98</td><td>0</td><td>12</td><td>0</td><td>10</td><td>0</td><td>1.85</td></tr>
<tr><td>19.07.2024 (5th)</td><td>0</td><td>80</td><td>0</td><td>3</td><td>0</td><td>22</td><td>0</td><td>1.48</td></tr>
<tr><td>29.07.2024 (6th) ***</td><td>0</td><td>0</td><td>0</td><td>0</td><td>0</td><td>0</td><td>0</td><td>0</td></tr>
</table>

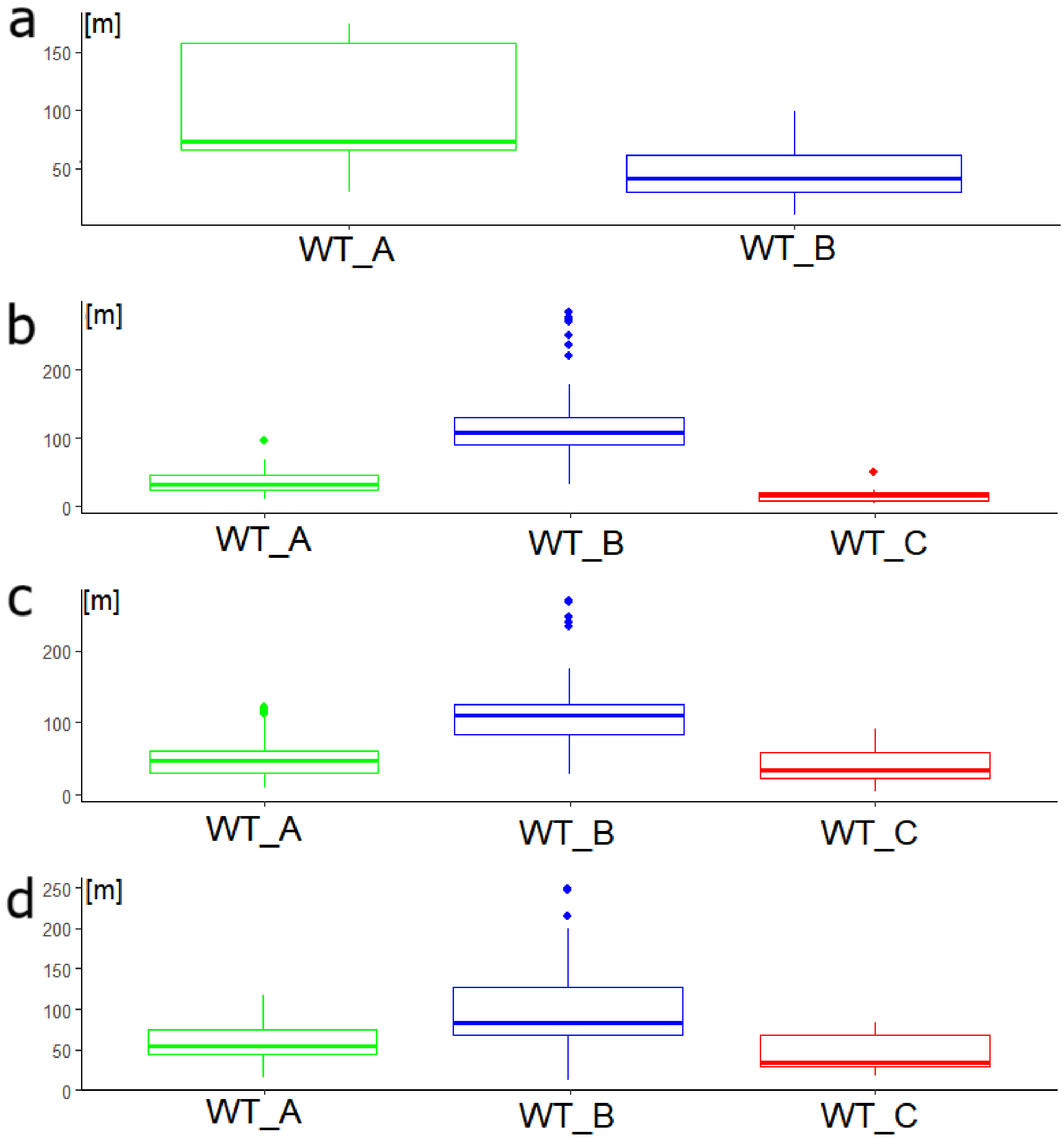

Figure S1_1. Distances between the particular nests and the geometrical colony center (centroid, DistCentr) in Whiskered Tern colonies: green – WT_A colony, blue – WT_B colony; red – WT_C colony, during subsequent visists in the field (a) – visit 2; (b) – visit 3; (c) – visit 4; (d) – visit 5. Boxplots indicate the median (band inside the box), the first (25%) and third (75%) quartile (box), the lowest and the highest values within 1.5 interquartile range (whiskers), and outliers (dots).

Table S1_2. Median and IQR of distances between nests and colony center (DistCentr) at observed Whiskered Tern Colonies (WT_A, WT_B, WT_C) during subsequent visists in the field.

Date (Number) of visit	Colony		
	WT_A (median ; IQR) [m]	WT_B (median ; IQR) [m]	WT_C (median ; IQR) [m]
04.06.2024 (2nd)	73.14 ; 92.37	41.33 ; 31.87	-
26.06.2024 (3rd)	31.74 ; 21.10	108.70 ; 39.84	16.25 ; 11.95
08.07.2024 (4th)	47.91 ; 31.49	109.68 ; 40.50	34.53 ; 36.42
19.07.2024 (5th)	54.38 ; 30.33	83.50 ; 57.86	34.27 ; 38.63

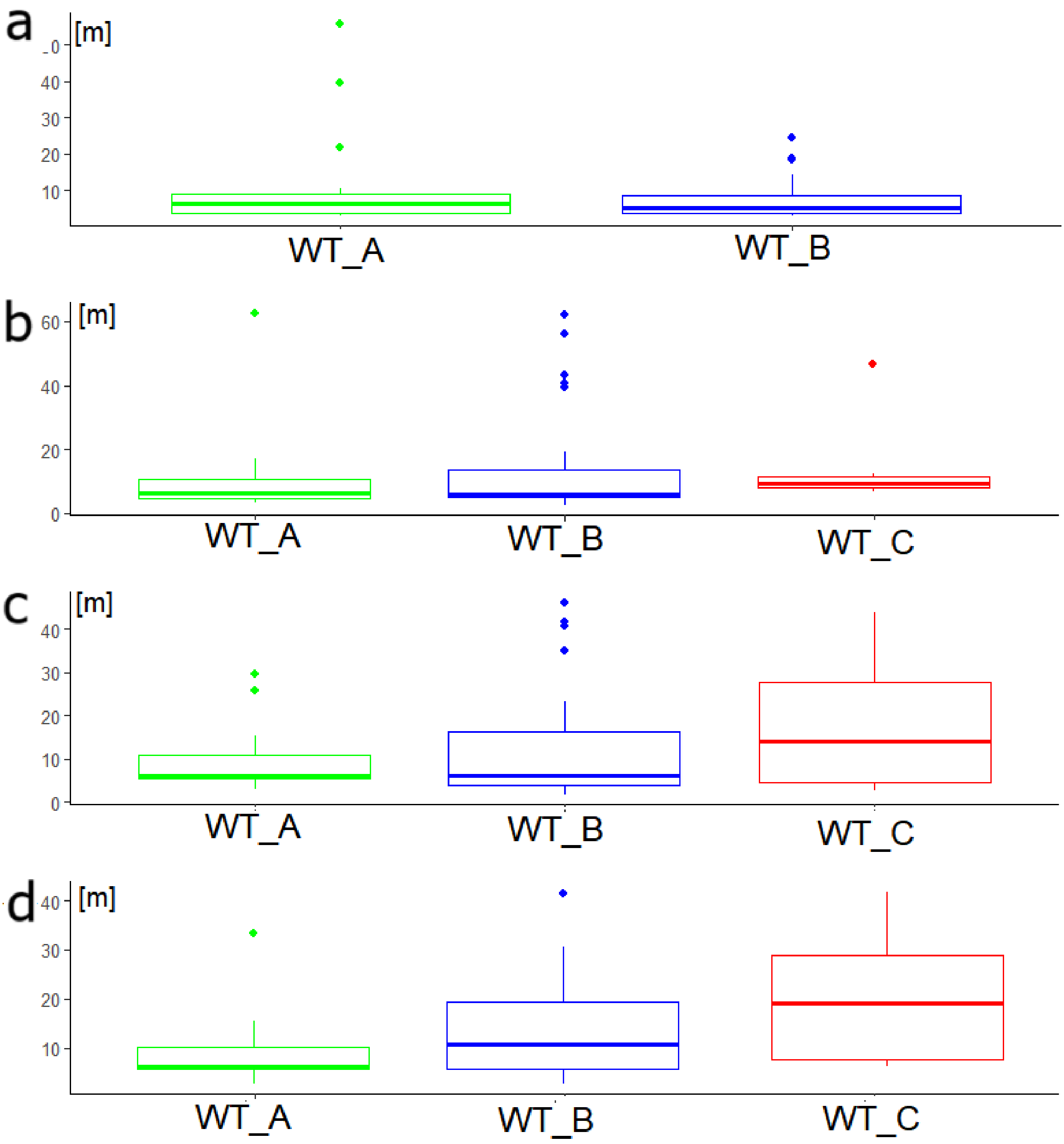

Figure S1_2. Distances between nests within particular colony (DistNest) in Whiskered Tern colonies: green – WT_A colony, blue – WT_B colony; red – WT_C colony, during subsequent visists in the field (a) – visit 2; (b) – visit 3; (c) – visit 4; (d) – visit 5. boxplots indicate the median (band inside the box), the first (25%) and third (75%) quartile (box), the lowest and the highest values within 1.5 interquartile range (whiskers), and outliers (dots).

Table S1_3. Median and IQR of distances nests within particular colony (DistNest) at observed Whiskered Tern Colonies (WT_A, WT_B, WT_C) during subsequent visists in the field.

Date (Number) of visit / Colony	WT_A (median ; IQR) [m]	WT_B (median ; IQR) [m]	WT_C (median ; IQR) [m]
04.06.2024 (2nd)	6.40 ; 5.41	5.34 ; 4.81	-
26.06.2024 (3rd)	6.51 ; 5.79	5.98 ; 8.80	9.34 ; 3.57
08.07.2024 (4th)	6.15 ; 5.53	6.28 ; 12.18	13.96 ; 23.13
19.07.2024 (5th)	6.16 ; 4.53	10.67 ; 13.57	19.18 ; 21.23

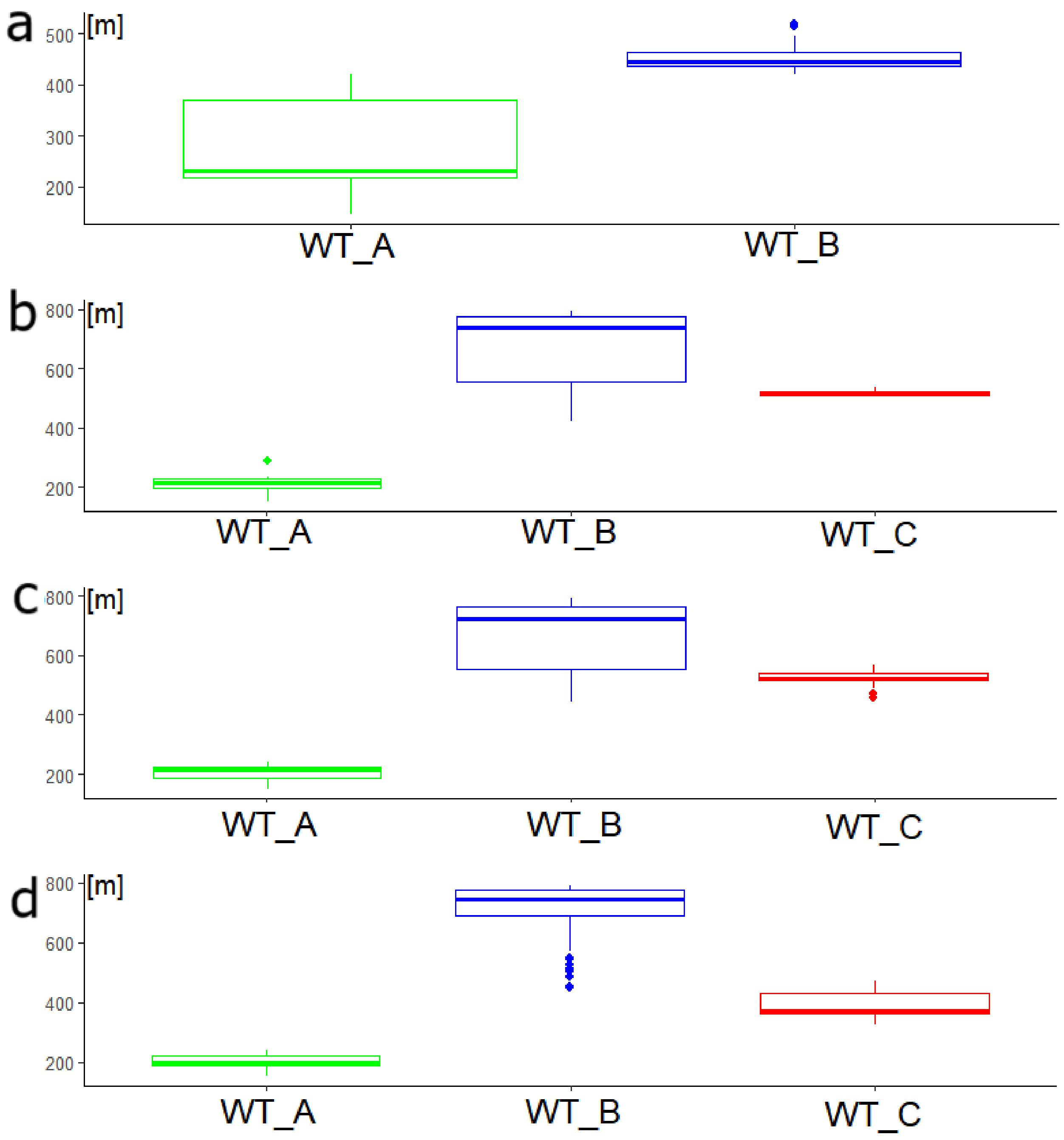

Figure S1_3. Distances between nests and waetrway Elbląg Canal (Dist_Canal) at observed Whiskered Tern Colonies: red – WT_1 colony, green – WT_2 colony; blue – WT_3 colony, during subsequent visists in the field (a) – visit 2; (b) – visit 3; (c) – visit 4; (d) – visit 5. Boxplots indicate the median (band inside the box), the first (25%) and third (75%) quartile (box), the lowest and the highest values within 1.5 interquartile range (whiskers), and outliers (dots).

Table S1_4. Median and IQR of distances between nests and Elbląg Canal (Dist_Canal) at observed Whiskered Tern Colonies (WT_1, WT_2, WT_3) during subsequent visists in the field.

Date (number) of visit	Colony		
	WT_A (median ; IQR) [m]	WT_B (median ; IQR) [m]	WT_C (median ; IQR) [m]
04.06.2024 (2nd)	232.32 ; 151.32	445.52 ; 25.82	-
26.06.2024 (3rd)	217.29.24 ; 32.46	738.40 ; 220.62	516.74 ; 11.74
08.07.2024 (4th)	212.33 ; 34.38	722.54 ; 213.55	522.06 ; 24.81
19.07.2024 (5th)	196.20 ; 31.54	744.74 ; 84.53	370.20 ; 69.10

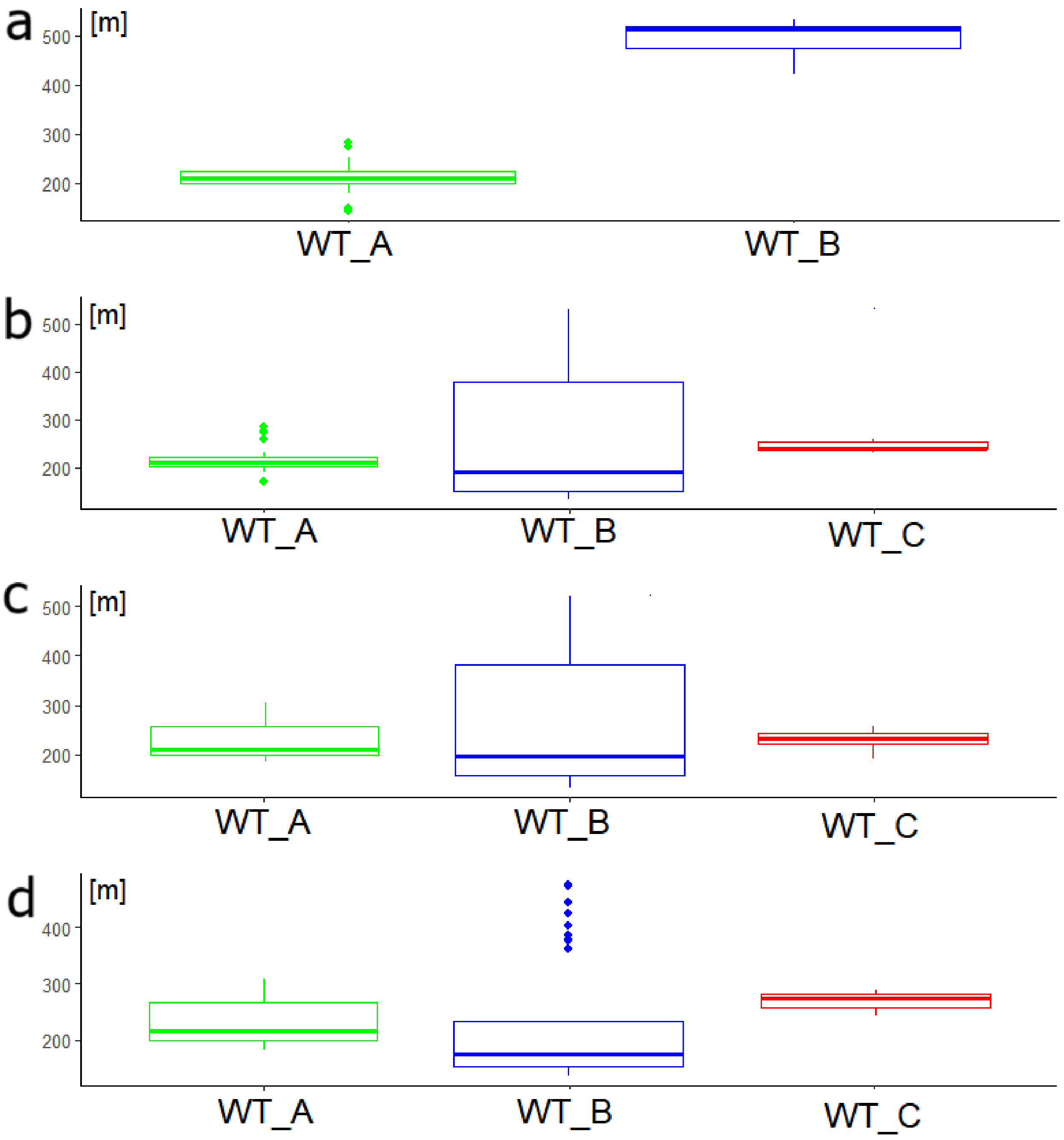

Figure S1_4. Distances between nests Druzno Lake shoreline (Dist_Shore) at observed Whiskered Tern Colonies: green – WT_A colony, blue – WT_B colony; red – WT_3C colony, during subsequent visists in the field (a) – visit 2; (b) – visit 3; (c) – visit 4; (d) – visit 5. Boxplots indicate the median (band inside the box), the first (25%) and third (75%) quartile (box), the lowest and the highest values within 1.5 interquartile range (whiskers), and outliers (dots).

Table S1_5. Median and IQR of distances between nests and Druzno Lake shoreline (Dist_Shore) at observed Whiskered Tern Colonies (WT_1, WT_2, WT_3) during subsequent visists in the field .

Date (Number) of visit	Colony		
	WT_A (median ; IQR) [m]	WT_B (median ; IQR) [m]	WT_C (median ; IQR) [m]
04.06.2024 (2nd)	211.35 ; 25.75	512.58 ; 44.29	-
26.06.2024 (3rd)	209.18 ; 19.89	190.62 ; 229.79	239.89 ; 15.96
08.07.2024 (4th)	210.13 ; 57.71	197.37 ; 222.50	233.41 ; 22.03
19.07.2024 (5th)	216.20 ; 66.59	175.93 ; 80.94	275.48 ; 24.34

Table S1_6. Differences in all considered colony measurements between WT colonies recorded during the consecutive visits. W - Wilcoxon test for 2nd visit with 2 colonies, χ^2 - Kruskal-Wallis test (for 3rd – 5th visits) values of. Significant differences are bolded.

Visit date	Dist_Nest	Dist_Shore	Dist_Centr	Dist_Canal
04.06	W = 261; P = 0.87; df = 2, 45	**W = 0; P < 0.001; df = 2, 45**	**W = 429; P < 0.001; df = 2, 45**	**W = 0; P < 0.001; df = 2, 45**
26.06	χ^2_2= 2.7846; P = 0.25	χ^2_2= 3.874; P = 0.14	**χ^2_2= 54.462; P < 0.001**	**χ^2_2= 60.577; P < 0.001**
08.07	χ^2_2= 3.0275; P = 0.22	χ^2_2= 1.5369; P = 0.46	**χ^2_2= 37.217; P < 0.001**	**χ^2_2= 71.206; P < 0.001**
19.07	**χ^2_2= 9.0092; P = 0.01**	**χ^2_2= 11.351; P = 0.003**	**χ^2_2= 17.961; P = 0.0001**	**χ^2_2= 63.341; P < 0.001**

Table S1_7. Nesting niche sizes – Resource niche deifned by NDVI (Normalized Difference Vegetation Index, proxy of vegetation density), NDWI (Normalized Difference Water Index, proxy of open water area contribution), Distance niche deifned by distances between neighbouring nests in colony (Dist_Nests), distances between nests and shoreline of the Druzno Lake (Dist_Shore), and distances between nests and Elbląg Canal (Dist_Canal). Nesting niche sizes reported as estimated values (EST) with standard error SE) for every Whiskered Tern (WT) colony during the consecutive visits.

Colony	Niche size (EST ± SE)			
	04.06 (2nd visit)	26.06 (3rd visit)	08.07 (4th visit)	19.07 (5th visit)
	Resource niche			
WT_A	(0.007± 0.002)	(0.0014723252 ± 0.0002708258)	(0.002117880 ± 0.000375826)	(0.0033172591 ± 0.0006073044)
WT_B	(0.0013± 0.0003)	(0.0024898706 ± 0.0003651587)	(0.0028808615 ± 0.0004142213)	(0.0021775363 ± 0.0003514481)
WT_C	-	(0.0002942766 ± 0.0001154992)	(0.0006790281 ± 0.0001711460)	(0.0012224917 ± 0.0004660088)
	Distance niche			
WT_A	(2,475,989.8 ± 650,871.7)	(359,025.78 ± 84,542.13)	(297,254.87 ± 62,259.28)	(315,548.83 ± 70,422.95)
WT_B	(110,743.13 ± 28,674.98)	(1138291 ± 209702)	(909,092.7 ± 151,805.4)	(407,436.33 ± 77,841.92)
WT_C	-	(54,123.25 ± 26,908.54)	(419,239.9 ± 127,252.4)	(144,892.55 ± 62,873.57)

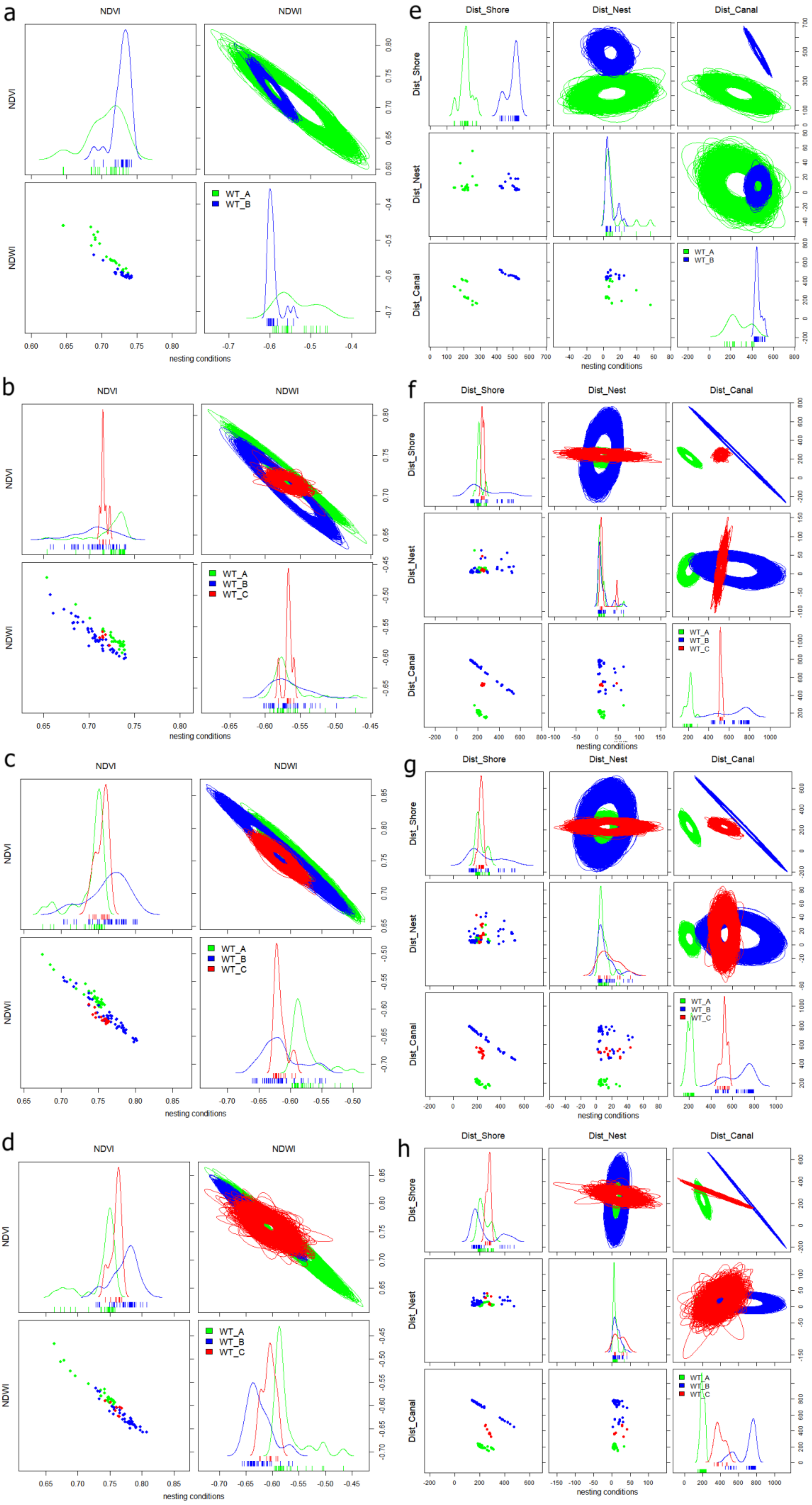

a
NDVI
NDWI
WT_A
WT_B
nesting conditions
b
NDVI
NDWI
WT_A
WT_B
WT_C
nesting conditions
c
NDVI
NDWI
WT_A
WT_B
WT_C
nesting conditions
d
NDVI
NDWI
WT_A
WT_B
WT_C
nesting conditions
e
Dist_Shore
Dist_Nest
Dist_Canal
WT_A
WT_B
nesting conditions
f
Dist_Shore
Dist_Nest
Dist_Canal
WT_A
WT_B
WT_C
nesting conditions
g
Dist_Shore
Dist_Nest
Dist_Canal
WT_A
WT_B
WT_C
nesting conditions
h
Dist_Shore
Dist_Nest
Dist_Canal
WT_A
WT_B
WT_C
nesting conditions

Figure S1_5. Visual projections of Resource (a-d) and Distance (e-h) nesting niches of WT colonies during 2nd (04.06.2024) (a-e), 3rd (26.06.2024) (b-f), 4th (08.07.2024) (c-g), and 5th (19.07.2024) (d-h) visit. Density plots indicate distribution of particular variables displayed as one-dimensional density plots; ellipses and two-dimensional scatterplots, representing (n = 10) randomly sampled projections of the nesting niches in two-dimensional perspectives defined by two environmental variables. Dist_Canal – distances between nests and Elbląg Canal, (Dist_Nest) – distances between nests themselves, Dist_Shore – distances between nests and Druzno Lake shoreline, NDVI – Normalized Difference Vegetation Index (proxy of vegetation density), NDWI – Normalized Difference Water Index (proxy of open water contribution).
